# Examination of the relationship between serum zinc levels and peripheral neuropathy induced by paclitaxel/carboplatin combination therapy in gynecological cancer patients

**DOI:** 10.20407/fmj.2024-013

**Published:** 2024-10-31

**Authors:** Yutaka Torii, Kana Naito, Junichi Takagi, Akira Yasue, Kazuhiko Tsukada, Takuma Fujii, Haruki Nishizawa

**Affiliations:** 1 Department of Obstetrics and Gynecology, Fujita Health University, School of Medicine, Toyoake, Aichi, Japan; 2 Department of Gynecology, Fujita Health University Okazaki Medical Center, Okazaki, Aichi, Japan

**Keywords:** Chemotherapy-induced peripheral neuropathy, Zinc, TC therapy

## Abstract

**Objectives:**

Chemotherapy-induced peripheral neuropathy (CIPN), a frequently occurring adverse event associated with paclitaxel/carboplatin (TC) combination therapy, causes limb pain and markedly reduces the patient’s quality of life. Since zinc has been reported to be associated with neuropathic pain, we investigated the relationship between CIPN due to TC therapy and serum zinc levels.

**Methods:**

The study included 13 patients with gynecological cancer whose serum zinc levels were measured before and during TC therapy. CIPN was classified into severity grades based on the Common Terminology Criteria for Adverse Events v5.0. A retrospective analysis was conducted on the relationship between the serum zinc level before TC therapy (PreZn), the minimum serum zinc level measured during TC therapy (MinZn), the MinZn/PreZn ratio, the number of TC treatment cycles, and the maximum grade of CIPN (MaxG) using Pearson’s correlation coefficient. Moreover, an analysis was also conducted on clinical factors influencing MaxG, as well as fluctuations in serum zinc levels and CIPN grades for each cycle of TC therapy.

**Results:**

A negative correlation was observed between the MinZn/PreZn ratio and MaxG (r=–0.557, p=0.048). The clinical factors influencing CIPN remained unclear, and the decrease in serum zinc levels and the aggravation of CIPN plateaued after the third cycle.

**Conclusions:**

If a decrease in serum zinc levels during TC therapy is smaller than before therapy, it may imply the existence of a causal relationship that suppresses the aggravation of CIPN.

## Introduction

Chemotherapy is used to treat many malignant tumors, but chemotherapy-induced peripheral neuropathy (CIPN) may occur as an adverse event of chemotherapy, which is frequently observed with the administration of medications such as paclitaxel, vincristine, and oxaliplatin. It poses a major problem as it significantly reduces the patient’s quality of life. Possible risk factors for the onset of CIPN include a history of diabetes and renal dysfunction.^1–3^

Paclitaxel/carboplatin (TC) therapy is the most commonly used first-choice chemotherapeutic agent in postoperative adjuvant therapy for gynecologic cancer and treatment for recurrent and advanced cases of such cancers. However, the incidence of CIPN caused by paclitaxel is high, and its etiological mechanism is not clearly understood, nor are there any clear therapeutic and preventive methods.^4,5^

Paclitaxel exerts its antitumor effects by promoting the polymerization of microtubules, stabilizing them to a high degree, and inhibiting depolymerization to suppress cell division.^6^ Zinc ions are contained in the junctions between α-tubulin subunits that constitute microtubules, and these ions are presumed to activate antitumor effects by contributing to stabilization. Zinc has also been reported to be involved in CIPN.^7^

Zinc is a metallic element known to mankind since antiquity. It is a constituent of more than 300 enzymes involved in cell replication, protein synthesis, and injury repair systems and is also known to have anti-inflammatory effects.^8^ Zinc is also implicated in neuropathic pain and thermal hyperalgesia, with there being sporadic reports of it exacerbating or alleviating symptoms in a dose-dependent manner.^9–12^ Research has suggested that zinc may have a significant effect on CIPN and neuropathic pain through the function of transient receptor potential vanilloid 1 (TRPV1), which is a capsaicin receptor. The possibility has been discussed that administrating zinc to patients undergoing chemotherapy may reduce the frequency and severity of such conditions.^13,14^

Therefore, we focused on the possibility that zinc may be involved in the onset and aggravation of CIPN associated with TC therapy. Based on previously published literature, we examined the fluctuations in serum zinc levels to elucidate the relationship.

## Methods

### Target Patients and Evaluation Methods

Of 29 patients with gynecological cancer who underwent initial postoperative TC therapy at our hospital from April 2022 to March 2024, 13 who had their serum zinc levels measured before and during TC therapy were investigated (Table 1). Zinc was measured using the colorimetric method, with reference values ranging from 80 to 130 μg/dL. Peripheral neuropathy was rated by two researchers based on the National Cancer Institute-Common Terminology Criteria for Adverse Events (NCI-CTCAE) v5.0, on a severity scale of grade 1–4 (grade 1: no symptoms or mild symptoms, grade 2: moderate symptoms or limitation of daily activities other than self-care, grade 3: severe symptoms or limitation of daily activities for self-care, and grade 4: life-threatening or requiring emergency treatment). We retrospectively examined the relationship between serum zinc level before TC therapy (PreZn), the minimum serum zinc level measured during TC therapy (MinZn), the MinZn/PreZn ratio as an indicator for the rate of decline in serum zinc levels before and during TC therapy, the number of TC therapy cycles, and the maximum grade of peripheral neuropathy that developed during TC therapy (MaxG). No restrictions were placed on supportive care for CIPN. TC therapy was administered at the most common dose and administration cycle for gynecologic cancers (paclitaxel 175 mg/m^2^ day 1, carboplatin AUC 6.0 day 1, 3-week intervals). The criteria for dose reduction or discontinuation due to adverse events were set as per known clinical trials and were applied when grade 4 or higher hematological toxicity and grade 3 or higher non-hematological toxicity occurred.

Moreover, the patients were classified into those with a MaxG of 1 (MaxG 1, cases 1–7) and those with a MaxG of 2 or higher (MaxG 2+, cases 8–13), and clinical factors of age, body mass index (BMI) (kg/m^2^), glycated hemoglobin (HbA1c) (National Glycohemoglobin Standardization Program) (%), and estimated glomerular filtration rate (eGFR) (ml/min/1.73 m^2^) were extracted to examine their possible influence on the onset or aggravation of CIPN. Furthermore, serum zinc levels were compared between the two MaxG groups for each cycle of TC therapy, and the transition of the average CIPN grade in the MaxG 2+ group was also examined.

### Statistical Analysis

EZR version 1.67 (https://www.jichi.ac.jp/saitama-sct/SaitamaHP.files/download.html) was used for all statistical analyses.^15^ EZR is statistical software that extends the functions of R and R Commander. Correlation analysis was performed using Pearson’s correlation coefficient, and the two groups were compared using the Mann–Whitney U test. A p-value of <0.05 on both sides was considered statistically significant.

### Research Ethics

This study was approved by the Medical Research Ethics Review Committee of the Fujita Health University School of Medicine as a comprehensive survey study (No. HM23-251). The research subjects were provided with information on the “Disclosure of Information on Medical Research Involving Human Subjects” section of the university’s website and were allowed to indicate their intention not to consent to the research using an opt-out format.

## Results

The median age of the patients was 61 (31–78) years. The primary disease was uterine cancer in eight patients, ovarian cancer in two patients, and peritoneal cancer in three patients. By disease stage, five patients were at Stage 1, two were at Stage 2, four were at Stage 3, and two were at Stage 4. Mecobalamin, pregabalin, and duloxetine were administered as supportive therapy for CIPN at the discretion of the attending physician. For cases 10 and 12, zinc acetate hydrate was prescribed for approximately 2 months after the final cycle of TC therapy as a treatment for taste disorder. No patients presented with peripheral neuropathy before the start of TC therapy or had a history of chemotherapy. Five patients (cases 1, 5, 11, 12, and 13) had either dose reduction or discontinuation during TC therapy, and two patients experienced dose reduction and discontinuation of TC therapy due to CIPN (Table 1, denoted by * and **).

The median values of PreZn and MinZn and the median number of cycles for TC therapy were 75 μg/dL (42–94 μg/dL), 65 μg/dL (34–99 μg/dL), and 6 (1–6), respectively, and MaxG was 1 in 7 cases, 2 in 3 cases, 3 in 2 cases, and 4 in 1 case. Serum zinc levels were below the reference range before the start of TC therapy in 9 patients (69%), and these levels further decreased with TC therapy. Additionally, four patients had a history of diabetes mellitus.

**Figure 1:**
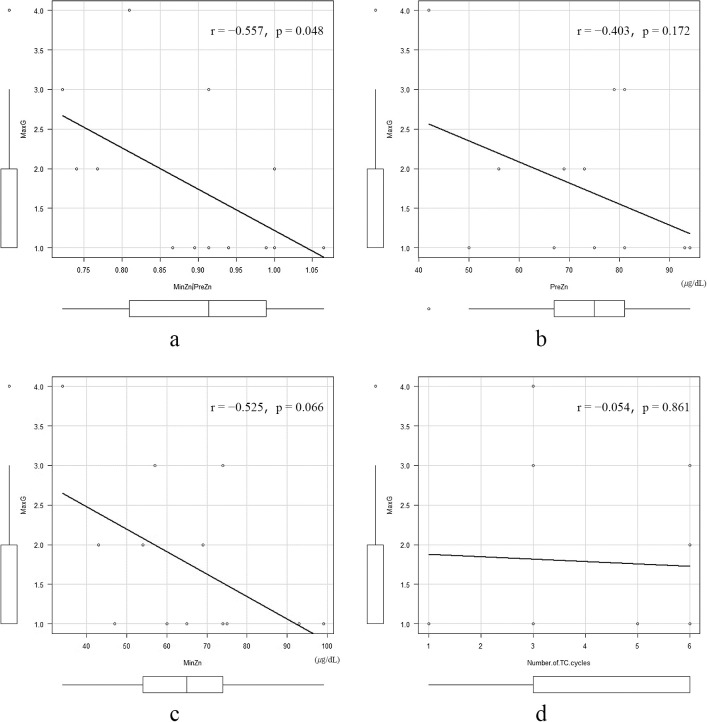
Correlation between serum zinc levels, the number of TC therapy cycles, and MaxG A significant negative correlation was found between the MinZn/PreZn ratio and MaxG, with more severe CIPN resulting in decreased serum zinc levels (r=–0.557, p=0.048). The following showed no correlation with the MaxG: b) PreZn (r=–0.403, p=0.172), c) MinZn (r=–0.525, p=0.066), and d) Number of TC therapy cycles (r=–0.054, p=0.861).

**Figure 2:**
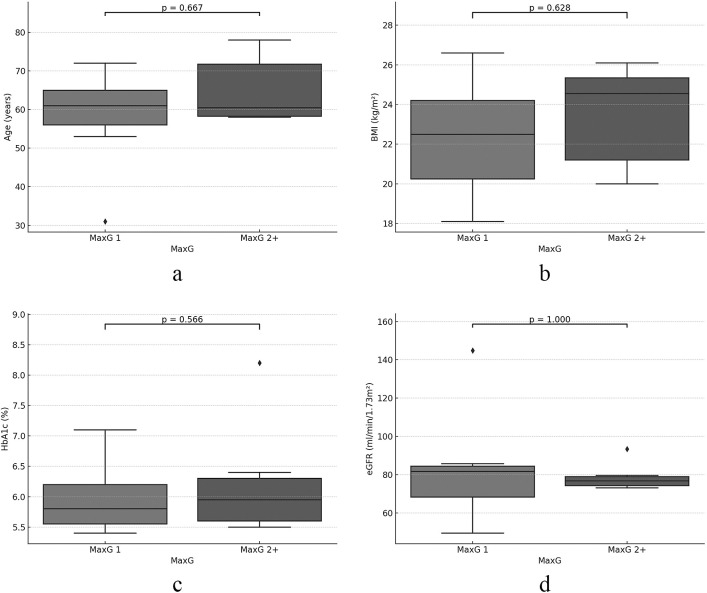
Examination of clinical factors when the patients were classified into MaxG 1 (MaxG 1 group) and MaxG 2 or higher (MaxG 2+ group) The following clinical factors did not affect the severity of CIPN: a) age (p=0.667), b) BMI (p=0.628), c) HbA1c (p=0.566), and d) eGFR (p=1.000).

Regarding the relationship with CIPN associated with TC therapy, a significant negative correlation was noted between the MinZn/PreZn ratio and MaxG (r=–0.557, p=0.048) (Figure 1a), while the correlation coefficients of the PreZn, MinZn, and number of cycles of TC therapy with the MaxG were –0.403 (p=0.172), –0.525 (p=0.066), and –0.054 (p=0.861), respectively (Figure 1b–d). In the two cases in which TC therapy was reduced and discontinued due to CIPN, the serum zinc level decreased from 81 μg/dL to 74 μg/dL on reduction or discontinuation of TC therapy compared to before (Case 11) and increased from 42 μg/dL to 48 μg/dL (Case 13), showing no consistency between the two cases. Age, BMI, HbA1c, and eGFR did not differ between the MaxG 1 and MaxG 2+ groups (Figure 2a–d). The presence or absence of diabetes mellitus was also investigated, but it had no effect (p=0.676).

**Figure 3:**
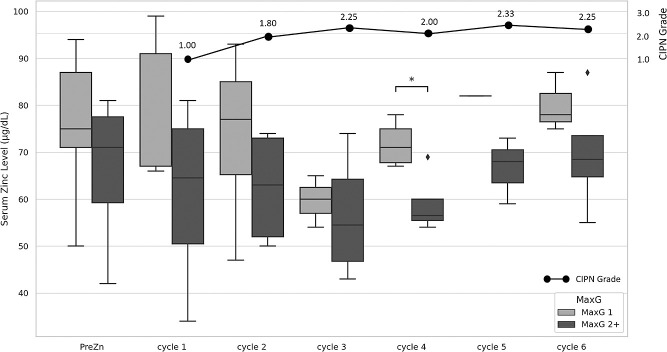
Fluctuations in serum zinc levels and average CIPN grades for each TC therapy cycle Serum zinc levels before and for each TC therapy cycle were classified as MaxG 1 (light gray) and MaxG 2+ (dark gray) and shown in a box diagram. The MaxG 2+ group showed lower serum zinc levels, exhibiting significantly lower serum zinc levels than the MaxG 1 group in cycle 4 (*: p=0.026). The line graph in the upper right direction shows the average CIPN grade for each cycle of TC therapy in the MaxG 2+ group. The CIPN grade increased with the number of TC therapy cycles, but it plateaued after the third cycle.

Figure 3 shows a comparison of serum zinc levels in the two MaxG groups for each cycle of TC therapy and the fluctuations in the mean CIPN grade in the MaxG 2+ group. The serum zinc levels tended to be lower in the MaxG 2+ group, and a significant difference was noted in the serum zinc levels between the two groups in cycle 4 (p=0.026). The CIPN grade also increased with the number of TC therapy cycles, but it stabilized at grade 2 to 2.3 from the third cycle onward. After the end of treatment, the CIPN grade decreased in all patients in the MaxG 2+ group and improved to grade 1 except for cases 12 and 13.

## Discussion

CIPN is caused by several chemotherapeutic agents, including paclitaxel, oxaliplatin, vincristine, cisplatin, and bortezomib. Although symptoms often improve after chemotherapy, some patients experience persistent symptoms. Factors that have been studied as causing CIPN include mitochondrial dysfunction, oxidative stress, the involvement of immune cells, and changes in ion channels in mouse models. Risk factors for the onset of CIPN include a history of neuropathic diseases, such as diabetes, before the start of chemotherapy, renal dysfunction accompanied by reduced creatinine clearance, and a history of smoking.^1,2^ However, no clear contributing factors were identified in this study. A meta-analysis showed that the prevalence of CIPN was 68.1%, 60.0%, and 30.0% at 1 month, 3 months, and 6 months after the end of chemotherapy, respectively. Many patients experience no improvement in their neurological symptoms even after the end of treatment, hence adversely affecting their quality of life.^3^

Several methods are available for assessing the severity of CIPN, including the Functional Assessment of Cancer Therapy/Gynecologic Oncology Group-Neurotoxicity (FACT/GOG-Ntx), which assesses patients by having them answer questionnaires, the European Organization for Research and Treatment of Cancer Quality of Life Queationnaire-CIPN20 (EORTC QLQ-CIPN20), and the NCI-CTCAE, in which medical professionals assess the patient’s condition.^16,17^ In this study, we selected the NCI-CTCAE as an assessment method that can be easily incorporated into daily practice and has high reproducibility. Although there are several reports on the validity or correlation of each assessment method, it is difficult to identify the most appropriate method to determine the severity of CIPN because of the differences in the agreement rate between the methods.^18–20^

Several clinical trials have been conducted to establish effective preventive or therapeutic methods for CIPN. Table 2 shows the main reports. Monosialotetrahexosylganglioside, a drug for treating diabetic peripheral neuropathy and Parkinson’s disease, topical ketamine and amitriptyline creams targeting multiple neuropathogenic mechanisms, adrenocorticotropic hormone derivatives, and oral lithium were not effective measures against CIPN.^21–25^ Alpha-lipoic acid, a type of fatty acid, has been shown to ameliorate peripheral neuropathy through its antioxidant effects. Although the therapeutic effects of cisplatin and oxaliplatin on CIPN are not consistent, it has been reported to inhibit CIPN progression in breast cancer patients receiving paclitaxel.^26–28^ Intravenous administration of glutathione, which has been shown to reduce neurotoxicity by decreasing platinum accumulation in the dorsal root ganglia, did not produce an effective therapeutic effect on CIPN associated with TC therapy.^29^ In a placebo-controlled, double-blind, randomized comparative trial, intravenous administration of calcium and magnesium to patients receiving oxaliplatin did not prove its efficacy against CIPN.^30^ On the other hand, there were reports that habitual magnesium intake contributed to a reduction in the prevalence and severity of CIPN.^31^ Other reports stated that vitamin E intake reduced the incidence of CIPN in some patients and that vitamin B intake decreased the severity of CIPN, although its incidence did not change.^32–34^ Despite these attempts to establish effective methods for CIPN using various approaches, the only drug presently recommended is duloxetine, a serotonin-noradrenaline reuptake inhibitor, and no definitive causative therapeutic modalities have been developed to prevent CIPN.^4,5,35^

In this study, we evaluated CIPN in patients with gynecological cancer who underwent TC therapy and discovered that not only paclitaxel but also carboplatin is a potential cause of peripheral neuropathy. Comparative studies of carboplatin in combination with pegylated liposomal doxorubicin and docetaxel have shown that CIPN caused by paclitaxel is significantly more severe than that caused by the other drugs.^36,37^ On the other hand, other reports have stated that the incidence of CIPN in breast cancer patients treated with carboplatin alone or in combination with paclitaxel is not significantly different compared with that of patients treated with paclitaxel alone.^38^ Therefore, although paclitaxel and carboplatin were cited as the major causative agents of CIPN in this study, since there are many reports on the mechanism, prevention, and treatment of CIPN associated with paclitaxel, this article will mainly discuss CIPN caused by paclitaxel.

Paclitaxel induces dose-dependent neuropathy, which is corroborated by pathological findings of sural nerve biopsies in patients receiving paclitaxel, exhibiting nerve fiber loss, axonal atrophy, and secondary demyelination.^1,39^ In the present study, CIPN tended to worsen until the third cycle of TC therapy, after which the condition plateaued at grade 2 to 2.3 (Figure 3). The reason why the aggravation in CIPN was suppressed may be that two of the three patients with CIPN grade 3 or higher discontinued TC therapy after three cycles, suggesting that the case in which the dose of TC therapy was reduced due to adverse events not derived from CIPN indirectly prevented CIPN from worsening. In addition, it has been investigated whether paclitaxel increases the expression of TRPV1, a capsaicin receptor, in the skin and dorsal root ganglion neurons, thereby aggravating CIPN.^40^ TRPV1 is an ionotropic receptor activated by endogenous substances and produced by nociceptive stimuli such as acid, heat, and inflammation. It is essential for expressing burning pain and hyperreflexia associated with inflammation in peripheral tissues and visceral organs and is believed to affect CIPN-related pain.^41–45^ TRPV1 is expressed in approximately 60% of nociceptors in the dorsal root and trigeminal ganglia and senses environmental stimuli in the skin and many visceral organs. It is significantly increased in the injured dorsal root ganglia and skin after nerve repair.^46,47^ In particular, TRPV1 has been reported to be involved in the cause of CIPN and is expected to be a major target in the development of new drugs to control pain in inflammatory diseases and neuropathic pain.^48^ One report demonstrated a relationship between paclitaxel-induced CIPN and TRPV1, showing that local administration of zinc dose-dependently suppressed paclitaxel-induced CIPN in mice. Since the effect of zinc administration in improving CIPN was significantly weakened in TRPV1-deficient mice, it is believed that zinc alleviates CIPN via the inhibition of TRPV1.^13^ These findings suggest that suppressing TRPV1 expression attenuates paclitaxel-induced CIPN and zinc may affect this process.

Many reports have stated that zinc is involved in CIPN. First, zinc has been shown to reduce pain by high-affinity binding to N-methyl-D-aspartate (NMDA) receptor subunits as a mechanism for neuropathic pain.^9^ Moreover, paclitaxel treatment reduces intravesicular zinc levels in hippocampal mossy fiber terminals and induces progressive cognitive dysfunction. This suggests that zinc may play an important role in the neurological complications of chemotherapy.^49^ Furthermore, zinc is present in dorsal root ganglion neurons, and depletion of vesicular zinc in the dorsal horn of the spinal cord enhances neuropathic pain in mice. Additionally, local administration of zinc to mice with sciatic nerve injury dose-dependently alleviates thermal hyperalgesia, suggesting a correlation between zinc and neuropathies.^11,12^ In the present study, a negative correlation was found between an increase in the MinZn/PreZn ratio, an index of the rate of decrease in serum zinc levels during TC therapy, and a decrease in MaxG. This was consistent with previous findings.

In a placebo-controlled, double-blind, randomized, controlled trial in which 55 cancer patients undergoing taxane treatment were assigned to take 25 mg of zinc sulfate orally once a day for 3 months as the intervention group, the frequency of CIPN was significantly reduced in the intervention group (14.8% vs. 37.03%, p<0.001).^14^ The severity of CIPN was also reduced in the NCI-CTCAE CIPN grading evaluation, and it has been stated that zinc administration may be an effective treatment for CIPN. However, the zinc dosage in this study was very low, approximately one twenty-sixth of the daily dose of zinc acetate hydrate commonly used for hypozincemia in Japan. Since the results require careful interpretation, it would be premature to conclude that zinc administration is effective. In another literature review looking at the effects of zinc intake, a review of oral zinc supplements mainly for head and neck cancer patients found no association with the occurrence or severity of oral mucositis, an adverse event of chemotherapy, but indicated that such zinc supplements may be effective against oral pain.^50^ Furthermore, there are reports that oral zinc supplements for non-chemotherapy-induced neuropathic pain have an analgesic effect due to their anti-inflammatory properties.^51^ Thus, although zinc administration may constitute a therapeutic method for CIPN, no conclusions have been reached. In the present study, since zinc acetate hydrate was administered to patients (cases 10 and 12) for taste disorders immediately after TC therapy, its effect on CIPN during the study period is considered negligible. Additionally, we observed a tendency for serum zinc levels to decrease and CIPN grade to increase as the number of cycles of TC therapy increased. Although no significant difference was noted in the correlation between the MinZn and MaxG, as shown in Figure 1c, they are considered candidate factors affecting CIPN. Therefore, it is hypothesized that maintenance of serum zinc levels may have an inhibitory effect on the severity of CIPN. However, supportive therapy for CIPN and patients’ learning of coping methods are thought to be confounding factors. Therefore, further research is needed to determine if zinc administration inhibits CIPN and clarify the significance of administering zinc before TC therapy.

When the action of zinc on the antitumor effect of chemotherapy is focused, there are generally positive reports. First, the cytotoxicity of zinc against ovarian cancer cell lines increases in a time- and concentration-dependent manner, suggesting that zinc may be used as a therapeutic agent for chemotherapy-resistant ovarian cancer.^52^ Second, studies on dietary intake of copper and zinc prior to ovarian cancer diagnosis and on the copper/zinc intake ratio and severity of ovarian cancer have shown that higher copper/zinc ratios correlate with lower degrees of severity of ovarian cancer, while no correlation was found with lower zinc intake amounts.^53^ In addition, it has been reported that zinc enhances and promotes sensitivity to paclitaxel in prostate cancer cells, that zinc levels are lower in prostate cancer tissues than noncancerous tissues, that zinc levels decrease with cancer progression, and that zinc may be used as an adjuvant to paclitaxel for prostate cancer.^54,55^ Breast and lung cancer patients also have lower serum zinc levels than noncancerous patients, and zinc deficiency is correlated with cancer severity and negatively correlated with survival.^56^ Tubulin, a component of microtubules that plays a key role in the antitumor effect of paclitaxel, exhibits impaired polymerization ability in zinc-deficient rats compared to control rats, suggesting that zinc may be involved in the antitumor effect of paclitaxel.^57^ On the other hand, excessive administration of zinc can cause myelopathy and peripheral neuropathy. As such, inappropriate administration should be strictly avoided.^58^

Nine of the thirteen patients (69%) in this study had hypozincemia (<80 μg/dL) before the start of TC therapy. Many cancer patients experience a decrease in blood zinc levels due to worsening nutritional status, inflammation and oxidative stress, and zinc consumption by cancer cells. In breast cancer patients, serum zinc levels decrease significantly as the disease stage progresses.^56^ In the present study, we classified the patients into 9 with PreZn <80 μg/dL and 4 with PreZn ≥80 μg/dL and investigated differences in the influence on disease stage. Although no significant difference was found, a correlation was suggested (p=0.0525). In addition, a comparison of PreZn levels between the MaxG 1 and 2+ groups showed no significant difference (p=0.316) (Figure 3), suggesting that the PreZn level decreases as the disease progresses. However, it is speculated that a decrease in serum zinc levels after the start of TC therapy may partially contribute to the worsening of CIPN. Zinc has shown cytotoxic and tumor-suppressing effects on cancer cells, making zinc supplementation a promising therapeutic option.

Our study found that serum zinc levels were less likely to decrease in patients with relatively mild CIPN during TC therapy, whereas serum zinc levels were more likely to decrease in patients with relatively severe CIPN, suggesting that these two phenomena are correlated. Since there have been reports of zinc administration achieving a CIPN improvement, a causal relationship may exist in which the worsening of CIPN is suppressed when a decrease in serum zinc levels is small. The limitations of this study are that it was a single-center study with a small number of patients, that it was not a prospective study with a clear definition of when serum zinc levels should be measured, and that 16 of the 29 TC-treated patients were excluded from the study because serum zinc levels were not systematically measured in all patients who had undergone TC. Moreover, given that the NCI-CTCAE, which was adopted as the evaluation method for CIPN, tends to underestimate the severity of CIPN compared to questionnaire-based evaluation methods such as FACT/GOG-Ntx and EORTC QLQ-CIPN20, the low reproducibility and inter-rater agreement may also pose a limitation to this study.^59^ Meanwhile, many unknowns remain about the pathogenesis of CIPN, the mechanism of its severity, and risk factors for its onset, and the most appropriate method for evaluating CIPN has not yet been established. Hence, the findings of this study may prove helpful to many patients who suffer from a decline in the quality of life due to CIPN. To determine whether zinc can be an effective treatment for CIPN, it is essential to consistently systematize the results of previous studies and proceed with clinical trials based on these results.

## Figures and Tables

**Table1:** List of eligible patients

Patient No.	Age (years)	Primary cancer	FIGO stage	MaxG (CTCAE v5.0)	PreZn (μg/dL)	MinZn (μg/dL)	MinZn/PreZn Ratio	Number of TC cycles	BMI (kg/m^2^)	History of diabetes	HbA1c (%)	eGFR (ml/min/1.73 m^2^)
1	72	Uterine	1	1	93	99	1.07	1*	25.6	+	6.5	64.1
2	61	Uterine	4	1	75	75	1	6	22.0		5.7	83.1
3	53	Uterine	1	1	75	65	0.87	3	18.5		5.4	81.7
4	64	Uterine	1	1	94	93	0.99	3	26.6		5.8	49.4
5	31	Ovarian	1	1	81	74	0.91	6*	18.1		5.4	85.7
6	66	Peritoneal	3	1	50	47	0.94	6	22.8	+	7.1	144.9
7	59	Peritoneal	3	1	67	60	0.90	5	22.5		5.9	72.5
8	58	Uterine	3	2	73	54	0.74	6	20.2		5.5	79.6
9	59	Uterine	1	2	69	69	1	6	24.9		6.0	73.4
10	75	Peritoneal	3	2	56	43	0.77	6	24.2	+	8.2	76.8
11	58	Uterine	2	3	81	74	0.91	3**	26.1	+	6.4	93.4
12	62	Uterine	2	3	79	57	0.72	6*	20.0		5.5	76.7
13	78	Ovarian	4	4	42	34	0.81	3**	25.5		5.9	73.1

*: dose reduction or/and discontinuation not caused by CIPN, **: dose reduction and discontinuation caused by CIPN

**Table2:** Clinical trials on CIPN treatment

References	Treatment for CIPN	Trials type	Sample size	Evaluation method	Endpoints	Chemotherapy	Outcomes
Wang 2020^23^	GM1, iv	RCT	196	NCI-CTCAE	Rate of grade 2 or worse cumulative neurotoxicity	FOLFOX	n.s. (GM1: 33.7% vs placebo: 31.6%, p=0.76)
Roberts 1997^24^	Hexapeptide of ACTH, iv	RCT	196	VPT	Percentage change in VPT	Cisplatin	n.s.
Koeppen 2004^25^	ACTH analogue, iv	RCT	147	13-item questionnaire	Neuropathy-free interval (the first occurrence of bilateral paresthesias)	Vincristine	n.s.
Gewandter 2014^26^	2% ketamine+4% amitriptyline cream	RCT	462	NRS	6-week NRS	Taxane 53%	n.s. (p=0.363)
Najafi 2021^27^	Lithium, po	RCT	36	decision by oncologist	Frequency of symptoms	Taxane	n.s. (p=0.352)
Guo 2014^28^	ALA, po	RCT	243	FACT/GOG-Ntx	FACT/GOG-Ntx scores at 24 weeks	Cisplatin or Oxaliplatin	n.s.
Werida 2022^29^	ALA, po	RCT	64	NCI-CTCAE	Grade comparison every three weeks	Paclitaxel	Significant improvement after the end of 9th and 12th weeks of paclitaxel intake (p=0.039)
Leal 2014^31^	Glutathione, iv	RCT	185	EORTC QLQ-CIPN20, NCI-CTCAE	Subscale of the EORTC QLQ-CIPN20 during the first 6 cycles of chemotherapy	TC	n.s. (EORTC QLQ-CIPN20: p=0.21, NCI-CTCAE: p=0.449)
Loprinzi 2014^32^	Calcium+Magnesium, iv	RCT	353	EORTC QLQ-CIPN20	Cumulative neurotoxicity	FOLFOX	n.s.
Wesselink 2018^33^	Calcium+Magnesium, po	Prospective cohort study	196	EORTC QLQ-CIPN20	The score before surgery, during chemotherapy and six months after chemotherapy	Oxaliplatin 85%	Dietary intake of magnesium during chemotherapy was associated with the prevalence of CIPN (PR 0.53, 95% CI 0.32, 0.90), and a higher dietary intake of magnesium was associated with less severe symptoms of CIPN
Kottscade 2011^34^	Vitamin E, po	RCT	207	NCI-CTCAE	Incidence of grade 2+	Taxane 58% Oxaliplatin 26%	n.s. (vitamin E: 34% vs placebo: 29%, p=0.43)
Chen 2021^35^	Vitamin E, po	meta-analysis	486	NSS, NDS, FGS	Incidense of CIPN	Taxane, Cisplatine, Oxaliplatin, others	Significantly reduced the incidence of CIPN (overall RR=0.55, 95% CI: 0.36, 0.85, p=0.007)
Schloss 2017^36^	Vitamin B, po	RCT	71	TNS	TNS assessed by an independent neurologist	Taxane, Oxaliplatin, Vincristine	n.s. in CIPN incidence (p=0.73) but reduced severity (12 weeks, p=0.03; 24 weeks, p=0.005; 36 weeks, p=0.021)

GM1: monosialotetrahexosylganglioside, RCT: randomized controlled trial, FOLFOX: fluorouracil+leucovorin+oxaliplatin, n.s.: no significant differences, ACTH: adrenocorticotropic hormone, VPT: vibration perception threshold, NRS: numeric rating scale, ALA: alpha-lipoic acid, NSS: neurological symptom score, NDS: neurological disability score, FGS: Hughes’ functional grading scale, TNS: total neuropathy score

## Supplementary Material

PDF-Japanese
