# Supplementary material for: Examination of the relationship between serum zinc levels and peripheral neuropathy induced by paclitaxel/carboplatin combination therapy in gynecological cancer patients
Source: Fujita Med J. 2024 Oct 31;11(1):11–9. doi: 10.20407/fmj.2024-013 (PMC11782941; doi:10.20407/fmj.2024-013)
Supplement: Supplementary file 1 — PDF-Japanese [file fmj-11-011-s001.pdf]

タイトル：婦人科癌におけるパクリタキセル/カルボプラチン併用療法誘発性末梢神経障害と血清亜鉛値の関連性に対する検討

ランニングタイトル：パクリタキセル/カルボプラチン誘発神経障害に対する亜鉛値の影響

Yutaka Torii, MD[1,2], Kana Naito, MD[1], Junichi Takagi, MD[1,2], Akira Yasue, MD, PhD[2], Kazuhiko Tsukada, MD, PhD[2], Takuma Fujii, MD, PhD[2], Haruki Nishizawa, MD, PhD[1]

[1]Department of Obstetrics and Gynecology, Fujita Health University, School of Medicine, Toyoake, Aichi, Japan

[2]Department of Gynecology, Fujita Health University Okazaki Medical Center, Okazaki, Aichi, Japan

Corresponding author: Yutaka Torii, MD

Department of Gynecology, Fujita Health University Okazaki Medical Center, 1, Gotanda, Harisaki-cho, Okazaki, Aichi 444-0827, Japan

Tel: 0564-64-8800, E-mail: y-torii@fujita-hu.ac.jp

抄録

目的：パクリタキセル/カルボプラチン（TC）併用療法に伴う有害事象で高頻度に発現する末梢神経障害（CIPN）は，四肢の疼痛を惹起し患者の QOL を著明に低下させる．亜鉛が神経因性疼痛に影響を及ぼす報告が散見されることから，TC 療法による CIPN と血清亜鉛値の関連性について検討した.

方法：TC 療法開始前と施行中に血清亜鉛値を測定した 13 例の婦人科癌患者を対象とした．CIPN は CTCAE v5.0 に基づいた重症度分類を行った．TC 療法前の血清亜鉛値（PreZn），TC 療法中に測定した血清亜鉛値の最低値（MinZn），MinZn/PreZn 比，TC 療法施行サイクル数と，CIPN の最高グレード（MaxG）との関連性についてピアソンの相関係数を用いて後方視的に解析を行った．また，MaxG に影響を及ぼす臨床学的因子を分析するとともに，TC 療法施行サイクル毎の血清亜鉛値および CIPN グレードの推移について検討した.

結果：MinZn/PreZn 比と MaxG に負の相関を認めた（r=-0.557，p=0.048）．他項目との相関性は認めなかった．CIPN に影響する臨床学的因子は明らかでなく，3 サイクル目以降で血清亜鉛値の低下と CIPN の重症化はプラトーとなった.

結論：TC 療法施行前と比較して施行中の血清亜鉛値の低下が少ない場合，CIPN の重症化を抑制する因果関係が存在している可能性が考えられる.

キーワード：化学療法誘発性末梢神経障害，亜鉛，TC 療法

序論

数多くの悪性腫瘍に対して化学療法が行われているが，化学療法の有害事象として発現する感覚性末梢神経障害（chemotherapy-induced peripheral neuropathy: CIPN）はパクリタキセル，ビンクリスチン，オキサリプラチンなどの薬剤投与により高頻度で認められ，患者の QOL を著しく低下させることが大きな問題となり，糖尿病の既往や腎機能障害などが発症リスク因子として考えられている．[1-3]

婦人科癌の術後補助療法や再発進行例に対する治療でパクリタキセル/カルボプラチン（TC）療法は第一選択として最も使用される化学療法薬である．しかしながら，パクリタキセルが引き起こす CIPN の頻度は高く，その発症機序は明確に解明されておらず，治療法および予防法も明らかでない．[4,5]

パクリタキセルは微小管の重合を促進し極度に安定化させ，脱重合を阻害して細胞分裂を抑制することにより抗腫瘍効果を発揮する．[6]　微小管を構成するα-チューブリンサブユニット間の接合部に亜鉛イオンが含まれており，安定化に寄与することで抗腫瘍効果へ影響を及ぼすと推察されているが，CIPN に対しても亜鉛が関与しているとの報告がみられる．[7]

亜鉛は古代から知られている金属元素で，細胞複製，タンパク質合成，傷害修復システムにおける 300 種類以上の酵素の構成素因であり，抗炎症作用を有することが知られてい

る．[8]　また，亜鉛は神経障害性疼痛や温痛覚過敏に関与するとされ，用量依存的に症状を増悪，または軽減する報告が散見される．[9-12]　亜鉛はカプサイシン受容体である transient receptor potential vanilloid 1（TRPV1）の機能を通じて CIPN および神経障害性疼痛に有意な影響を及ぼす可能性が示されており，化学療法を受けている患者に亜鉛を投与することで，その頻度や重症度が低下する可能性が議論された．[13,14]

　そこで，TC 療法に伴う CIPN の発現と重症化に亜鉛が関与している可能性に着目し，既報の文献レビューを行った上で，血清亜鉛値の推移を調査することにより，その関連性を検証した.

方法

対象患者と評価法

2022 年 4 月から 2024 年 3 月に当院で術後初回治療として TC 療法を行った婦人科癌患者 29 例中，TC 療法開始前と施行中に血清亜鉛値を測定した 13 例を対象とした（Table 1）．亜鉛の測定方法は比色法であり，基準値は 80～130 µg/dL である．末梢神経障害は NCI-CTCAE（National Cancer Institute-Common Terminology Criteria for Adverse Events） v5.0 に基づいてグレード 1 から 4 までの重症度分類の評価を 2 名で行った（グレード 1：無症状または軽度の症状，グレード 2：中等度の症状，または身の回り以外の日常生活動作制限，グレード 3：高度の症状，または身の回りの日常生活動作制限，グレード 4：生命を脅かす，緊急処置を要する）．TC 療法前の血清亜鉛値（PreZn），TC 療法中に測定した血清亜鉛値の最低値（MinZn），TC 療法前と施行中の血清亜鉛値の低下率の指標として MinZn/PreZn 比，TC 療法施行サイクル数と，TC 療法中に発症した末梢神経障害の最高グレード（MaxG）との関連性について後方視的に検討した．CIPN に対する支持療法の制約は設けていない．TC 療法は婦人科癌において最も一般的な投与量，投与サイクル（パクリタキセル 175mg/m^2 day1，カルボプラチン AUC 6.0 day1，3 週間隔）で行われ，有害事象による減量または中止の基準は既知の臨床試験に基づいて設定されており，Grade4 以上の血液毒性，または Grade3 以上の非血液毒性が発現した場合に適用された．

また， MaxG が 1 の群（MaxG 1，症例 1～7）と 2 以上の群（MaxG 2+，症例 8～13）に分類し，CIPN の発症または重症化へ影響を及ぼす可能性を検討するため，年齢，BMI (kg/m^2)，HbA1c (NGSP) (%)，eGFR (ml/min/1.73m^2)の臨床学的因子を抽出した．さらに，TC 療法施行サイクル毎の MaxG の 2 群における血清亜鉛値の比較と，MaxG 2+群の CIPN グレード平均値の推移についても検討した.

統計学的解析

すべての統計解析には EZR version 1.67（https://www.jichi.ac.jp/saitama-sct/SaitamaHP.files/download.html）を使用した．[15] EZR は R および R コマンダーの機能を拡張した統計ソフトウェアである．尚，相関分析はピアソンの相関係数，2 群間の比較はマン・ホイットニーU 検定を用いて行い，p 値は両側で 0.05 未満を統計学的有意とした.

研究倫理

本研究は包括的な調査研究として藤田医科大学医学部医学研究倫理審査委員会の承認を得た（番号 HM23-251）．研究対象者に対して，藤田医科大学医学部ホームページの「人を対象とする医学系研究に関する情報公開」で情報提供を行い，オプトアウト形式による

研究に許諾しない意思を示す機会を設けた．

結果

対象症例の年齢中央値は61歳（31～78歳），原疾患は子宮体癌が8例，卵巣癌が2例，腹膜癌が3例であり，病期はstage 1が5例，2が2例，3が4例，4が2例であった．CIPNに対する支持療法として，メコバラミン，プレガバリン，デュロキセチンが主治医判断で投与されていた．また，症例10，12は味覚障害に対する治療として，TC療法最終サイクル施行後に酢酸亜鉛水和物が約2か月間処方されていた．末梢神経障害をTC療法開始前から呈していた症例，および化学療法施行歴を有する症例は認めなかった．TC療法施行中に減量または中止を行った症例は5例（症例1, 5, 11, 12, 13）であり，CIPNに起因するTC療法の減量および中止を行ったのは2例であった（Table 1，*および**で表記）．

PreZn，MinZn，TC療法施行サイクル数の中央値はそれぞれ，75 ㎍/dL（42～94 ㎍/dL），65 ㎍/dL（34～99 ㎍/dL），6回（1～6回）であり，MaxGは，1が7例，2が3例，3が2例，4が1例であった．TC療法開始前から血清亜鉛値が基準値を下回っている症例が9例（69%）と多く，TC療法の施行に伴いその値はさらに低下した．また，糖尿病の既往がある症例を4例認めた．

TC療法に伴うCIPNとの関連性の検討では，MinZn/PreZn比とMaxGに有意な負の相関を認めた（r=-0.557，p=0.048）（Figure 1a）．PreZn，MinZn，TC療法施行サイク

ル数とMaxGとの相関係数はそれぞれ，-0.403 (p=0.172)，-0.525 (p=0.066)，-0.054 (p=0.861)であり関連性を認めなかった（Figure 1b-d）．また，CIPNが原因でTC療法が減量および中止となった2症例において，TC療法開始前と減量または中止時点での血清亜鉛値は，症例11は81 ㎍/dLが74 ㎍/dLへ低下，症例13は42 ㎍/dLが48 ㎍/dLへ増加しており，その増減に一貫性を認めなかった.

年齢，BMI，HbA1c，eGFRはMaxG 1と2+の2群間で差を認めなかった（Figure 2a-d）．また糖尿病の有無についても検討を行ったが，同様に影響を及ぼさなかった（p=0.676）.

TC療法施行サイクル毎のMaxGの2群における血清亜鉛値の比較と，MaxG 2+群のCIPNグレード平均値の推移についてFigure3に示す．MaxG 2+群において血清亜鉛値が低値である傾向がみられ，サイクル4では2群間の血清亜鉛値に有意差を認めた（p=0.026）．また，TC療法施行サイクル数増加に伴いCIPNグレードも上昇したが，3サイクル目以降はグレード2～2.3で安定化した．治療終了後はMaxG 2+群の全例でCIPNグレードは軽減し，症例12，13以外はグレード1まで改善した.

考察

CIPN はパクリタキセル，オキサリプラチン，ビンクリスチン，シスプラチン，ボルテゾミブなど，いくつかの化学療法剤によって引き起こされ，化学療法後に軽快することが多いが一部の患者は症状が持続する．CIPN の要因として，マウスモデルにおけるミトコンドリア機能障害，酸化ストレス，免疫細胞の関与やイオンチャネルの変化などが研究されており，発症リスク因子として，糖尿病など化学療法開始前の神経障害の既往，クレアチニンクリアランスの低下を伴う腎機能障害，喫煙歴が挙げられるが，本研究では明らかな要因は同定されなかった．[1,2] メタアナリシスによる CIPN の有病率は，化学療法投与終了後 1 か月，3 ヶ月，6 か月でそれぞれ 68.1%，60.0%，30.0%とされ，多くの患者が治療終了後も神経症状が軽快しないため QOL に悪影響を及ぼす．[3]

CIPN の重症度の評価方法は，質問票に患者が回答することで評価を行う FACT/GOG-Ntx（Functional Assessment of Cancer Therapy/Gynecologic Oncology Group-Neurotoxicity），EORTC QLQ-CIPN20（European Organization for Research and Treatment of Cancer Quality of Life Queationnaire-CIPN20），および医療者側が患者の状態を評価する NCI-CTCAE など複数存在する．[16,17] 本研究では，CIPN を日常診療で簡便に評価可能であり再現性が高い評価法として NCI-CTCAE を選択した．それぞれの評価法の妥当性，または相関性についての報告がいくつか認められるが，評価法間での一致率に差が見られることから，CIPN の重症度の判定に最も適切な手法を特定することは

困難である．[18-20]

CIPN に有効な予防法，または治療法の構築のため，いくつかの臨床試験がなされてきた．その主な報告を Table 2 に示す．糖尿病性末梢神経障害やパーキンソン病の治療薬であるモノシアロテトラヘキソシルガングリオシド，複数の神経障害誘発機序を標的とするケタミンおよびアミトリプチリンの外用クリームの塗布，副腎皮質刺激ホルモン誘導体，リチウム内服は CIPN に対する有効な手段とならなかった．[21-25] 脂肪酸の一種であるアルファリポ酸は抗酸化作用によって末梢神経障害を改善させる効果が示されており、シスプラチン，オキサリプラチンによる CIPN に対する治療効果は一定でないものの，乳がん患者に対するパクリタキセル投与中の CIPN の進行を抑制したとの報告がある．[26-28] 後根神経節内での白金の蓄積を減少させることで神経毒性を軽減する作用が示されているグルタチオンの静脈内投与により，TC 療法に伴う CIPN に有効な治療効果は発揮されなかった．[29] オキサリプラチン投与患者を対象としたカルシウム，マグネシウムの静脈内投与によるプラセボ対照二重盲検ランダム化比較試験では CIPN に対する有効性は証明されなかった．[30] その一方で，マグネシウムの習慣的摂取により CIPN の有病率，および重症度の低下に寄与したとの報告がみられた．[31] また，ビタミン E の摂取によって CIPN の発生率が一部で減少したという報告や，ビタミン B の摂取により CIPN の発生率は変わらないものの，重症度が減少したとする報告もみられる．[32-34] このように様々なアプローチにより CIPN に対して有効な手法の構築が試みられてきたにも関わらず，現時点で治療

薬として推奨されている薬剤はセロトニン・ノルアドレナリン再取り込み阻害薬であるデュロキセチンのみであり，予防するための明確な原因治療は開発されていない．[4,5,35]

今回我々はTC療法を行った婦人科癌患者のCIPNに関する評価を行っており，パクリタキセルだけでなくカルボプラチンも末梢神経障害の潜在的な原因として考えられる．カルボプラチンとの併用療法におけるリポソーマルドキソルビシンやドセタキセルとの比較研究では，パクリタキセルによるCIPNはこれら他薬剤より顕著に重篤であることが示されている．[36, 37] 一方で，乳癌患者に対するカルボプラチン単独投与またはパクリタキセルとの併用投与におけるCIPN発生率は，パクリタキセル単独投与と比較して有意差がないとする報告もある．[38] これらから，本研究におけるCIPNの主要な原因薬剤としてパクリタキセルとカルボプラチンの両剤が挙げられるものの，パクリタキセルに伴うCIPNの機序，予防や治療に関する報告が多数存在するため，本稿では主にパクリタキセルによるCIPNについて論じる．

パクリタキセルは投与回数依存性の神経障害を発症し，これはパクリタキセル投与患者の腓腹神経生検による神経線維減少，軸索萎縮，二次的脱髄を示す病理学的所見によって裏付けられている．[1,39] 本研究ではTC療法の3サイクル目まではCIPNの増悪傾向を示したが，以降はグレード2～2.3でプラトーとなった（Figure 3）．CIPNの悪化が抑制された原因として，CIPNグレード3以上の3症例中2症例がTC療法を3サイクルで中止となっていること，CIPNに起因しない有害事象によってTC療法の減量を行った症例が，

間接的に CIPN の増悪を予防した可能性が考えられる．また，パクリタキセルは皮膚と後根神経節ニューロンにおけるカプサイシン受容体である TRPV1 の発現を増加させ，CIPN を増強させる可能性が検討されている．[40] TRPV1 は酸，熱などの侵害刺激や炎症時に産生される内因性物質によって活性化されるイオンチャネル型受容体であり，末梢組織や内臓の炎症に伴う灼熱痛や反射亢進の発現に不可欠であり，CIPN 関連の痛みに影響しているとされる．[41-45] TRPV1 は後根神経節と三叉神経節にある侵害受容器の約 60%で発現しており，皮膚や多くの内臓器官で環境からの刺激を感知しており，傷害を受けた後根神経節や神経修復後の皮膚で有意に増加する．[46,47] 特に，CIPN の原因に TRPV1 は関与しているとの報告もあり，炎症性疾患における疼痛や神経障害性疼痛を制御する新規薬剤の開発における主要な標的として期待されている．[48] パクリタキセルによる CIPN と TRPV1 の関連性を示したものとして，亜鉛を局所投与することで用量依存的にマウスのパクリタキセルに伴う CIPN が抑制されたとする報告があり，TRPV1 欠損マウスでは亜鉛投与による CIPN 改善効果が著明に減弱したことから，TRPV1 の阻害を介して亜鉛が CIPN を軽減したとされる．[13] これらより，TRPV1 の発現を抑制することでパクリタキセルに伴う CIPN を減弱させるものと推察され，亜鉛がその過程に影響を与えている可能性を窺わせる．

CIPN に亜鉛が関与しているとする報告は多数みられる．まず，亜鉛の神経障害性疼痛に対するメカニズムとして，N-メチル-D-アスパラギン酸（NMDA）受容体のサブユニッ

トへの高親和性結合により痛みを軽減することが示されている．[9] また，パクリタキセル投与により海馬の苔状繊維終末の小胞内亜鉛濃度を低下させ，進行性の認知機能障害を引き起こすことから，亜鉛が化学療法の神経合併症における重要な役割を担っている可能性がある．[49] さらに，亜鉛は後根神経節ニューロンに存在しており，脊髄後角の小胞亜鉛が枯渇するとマウスの神経障害性疼痛が増強すること，坐骨神経損傷マウスに亜鉛の局所投与を行うと用量依存的に温痛覚過敏を緩和することからも亜鉛と神経障害の関連性が示唆される．[11,12] 本研究においては，TC 療法中の血清亜鉛値の低下率を示す指標としたMinZn/PreZn 比の上昇に伴い，MaxG が低下するという負の相関を認めたことは既報の知見と合致する．

タキサン治療中のがん患者 55 名に対して，硫酸亜鉛 25mg/日を 1 日 1 回 3 か月間内服した群を介入群としたプラセボ対照二重盲検ランダム化比較試験では，介入群で CIPN の頻度が有意に減少した（14.8% vs 37.03%, $p<0.001$）．[14] NCI-CTCAE による CIPN のグレード評価で重症度も低下を認めており，亜鉛の投与が CIPN に対する有効な治療法となり得る可能性が述べられている．しかしながら，この研究における亜鉛の投与量は本邦で低亜鉛血症に一般的に使用される酢酸亜鉛水和物の一日用量と比較して約 26 分の 1 と非常に低用量に設定されており，得られた結果の解釈には慎重を要するため，亜鉛投与が効果的であると結論付けることは早計と思われる．また，亜鉛摂取による効果を見た別の文献では，主に頭頸部がん患者を対象とした亜鉛サプリメント内服に関するレビ

ューで，化学療法の有害事象である口腔粘膜炎の発生，重症度に関与は認めなかったものの，口腔痛に対しては有効である可能性が示されている．[50] 一方で，化学療法誘発性ではない神経障害性疼痛に対する亜鉛サプリメント内服により抗炎症作用に起因した鎮痛効果を発揮したとする報告もみられる．[51] このように，亜鉛の投与が CIPN の治療法となる可能性はあるものの結論には至っておらず，本研究で味覚障害のために酢酸亜鉛水和物を使用した症例 10，12 は TC 療法終了直後に投与されているため，本研究対象期間中の CIPN に対する影響は極めて少ないと考えられる．本研究では TC 療法施行サイクル数の増加に伴って血清亜鉛値の低下，および CIPN グレードが上昇する傾向が観察されたこと，Figure1c に示す通り MinZn と MaxG の相関性に有意差は認められなかったものの，CIPN へ影響する因子の候補として挙げられることから，血清亜鉛値の維持が CIPN の重症度へ抑制的に働く仮説が導き出される．しかしながら，CIPN に対する支持療法や患者自身による対処法の習得などが交絡因子として考えられること，さらに亜鉛の投与が CIPN に対する抑制効果を持つと仮定した場合，TC 療法施行前から投与する必要性に関して等，不明な点が数多いことからさらなる詳細な研究による解明が必要と考えられる．

ここで，亜鉛の化学療法における抗腫瘍効果への影響に焦点を当てた場合，総じて肯定的な報告が散見される．まず，卵巣癌細胞株に対して，亜鉛の殺細胞性が時間と濃度に依存して高まることから，亜鉛は化学療法抵抗性卵巣癌に対する治療薬となる可能性が示されている．[52] 次に，卵巣癌診断前の銅，亜鉛の食事からの摂取量，および銅/亜鉛摂取量

の比と卵巣癌の重症度に関しての研究では，銅/亜鉛比の高値は卵巣癌の重症度低下と相関するとあるが，亜鉛の摂取量が低いことには関与しない．[53]　さらに，前立腺癌細胞では亜鉛がパクリタキセルに対する感受性を増強，促進するとされ，前立腺癌組織では非癌組織と比較して亜鉛濃度が低下しており，亜鉛濃度は癌の進行とともに減少することから，亜鉛は前立腺癌に対するパクリタキセルの補助療法となる可能性について述べられている．[54,55]　乳癌，肺癌患者でも同様に非癌患者と比較して血清亜鉛値が低く，亜鉛欠乏は癌の重症度と相関し，生存率と負の相関を示すとされる．[56]　パクリタキセルが抗腫瘍効果を発揮する主座たる微小管を構成するチューブリンは，亜鉛欠乏ラットにおいて対象ラットと比較して重合能に障害を示し，亜鉛投与により重合を刺激した研究結果からも，パクリタキセルの抗腫瘍効果に亜鉛が関与している可能性が示唆される．[57]　その一方で，亜鉛の過剰投与により脊髄症，末梢神経障害を引き起こすことがあり，不適切な投与は厳に避けるべきである．[58]

本研究における対象症例 13 例中 9 例（69%）が，TC 療法開始前から低亜鉛血症（＜80 µg/dL）を呈していた．多くの癌患者は栄養状態の悪化，炎症と酸化ストレス，癌細胞による亜鉛の消費などによって血中亜鉛レベルが低下するとされ，乳癌患者では病期の進行に伴い血清亜鉛値は有意に低下する．[56]　そこで，PreZn が 80 µg/dL 未満の 9 例と 80 µg/dL 以上の 4 例に分類し，病期に対する影響の有無を検討したところ，有意な差を認めなかったものの関連性が示唆された（p=0.0525）．また，MaxG 1 群と 2+群で PreZn を

比較検討した結果，有意差を認めなかったことから（p=0.316）（Figure 3），病勢の進行によって PreZn が低値となることが考えられるものの，CIPN が重症化する要因の一端として TC 療法開始後の血清亜鉛値の低下が影響していると推測される．亜鉛はがん細胞に対する細胞毒性と腫瘍抑制能力を示していることから亜鉛補充が治療の一環として有望であるとされている．

我々の研究により，TC 療法施行中の CIPN が比較的軽度の症例では血清亜鉛値の低下が少なく，CIPN が比較的高度の症例では血清亜鉛値の低下が多いという二つの事象が相関している結果を得た．亜鉛の投与により CIPN が改善した報告もあることから，血清亜鉛値の低下が少ない場合，CIPN の重症化を抑制している因果関係が存在している可能性が考えられる．本研究の課題として，少数例かつ単施設での検討であり，血清亜鉛値測定時期の明確な規定のある前方視的研究でないこと，TC 療法施行患者全例で系統的に血清亜鉛値を測定していないために，TC 療法を行った 29 例中 16 例が研究対象外となったことが挙げられる．また CIPN の評価法として採用した NCI-CTCAE は，FACT/GOG-Ntx や EORTC QLQ-CIPN20 などのアンケート型評価法と比較すると，CIPN の重症度が過小評価される傾向にあるため，再現性や評価者間での一致率の低さも本研究の課題である [59]。一方で、CIPN の発症機序，重症化のメカニズムや発症リスク因子は依然として不明な部分が多く，また CIPN の評価法として最も適切な手法も確立されていないため、CIPN による QOL 低下に悩む多数の患者に対して本研究の知見が貢献する可能性がある．亜鉛が

CIPN に対する有効な治療法となり得るかの検討にあたり，これまでの研究成果について整合性を持って体系化し，それをもとに臨床試験に進む方針が不可欠であると考えられた．

利益相反

著者らに開示すべき利益相反はない．

引用文献

1. Addington J, Freimer M. Chemotherapy-induced peripheral neuropathy: an update on the current understanding. F1000Res 2016; 5: F1000 Faculty Rev-1466.
2. Flatters SJ, Dougherty PM, Colvin LA. Clinical and preclinical perspectives on Chemotherapy-Induced Peripheral Neuropathy (CIPN): a narrative review. Br J Anaesth 2017; 119: 737–49.
3. Seretny M, Currie GL, Sena ES, Ramnarine S, Grant R, MacLeod MR, Colvin LA, Fallon M. Incidence, prevalence, and predictors of chemotherapy-induced peripheral neuropathy: A systematic review and meta-analysis. Pain 2014; 155: 2461-70.
4. Hershman DL, Lacchetti C, Dworkin RH, et al. Prevention and management of chemotherapy-induced peripheral neuropathy in survivors of adult cancers: American Society of Clinical Oncology clinical practice guideline. J Clin Oncol 2014; 32: 1941–67.
5. Jordan B, Jahn F, Sauer S, Jordan K. Prevention and Management of Chemotherapy-Induced Polyneuropathy. Breast Care (Basel) 2019; 14: 79–84.
6. Jordan MA, Wilson L. Microtubules as a target for anticancer drugs. Nat Rev

Cancer 2004; 4: 253–65.

7. Lowe J, Li H, Downing KH, Nogales E. Refined structure of αβ-tubulin at 3.5 Å resolution. J Mol Biol 2001; 313: 1045-57.

8. Lansdown AB, Mirastschijski U, Stubbs N, Scanlon E, Agren MS. Zinc in wound healing: Theoretical, experimental, and clinical aspects. Wound Repair Regen 2007; 15: 2–16.

9. Nozaki C, Vergnano AM, Filliol D, Ouagazzal AM, Le Goff A, Carvalho S, Reiss D, Gaveriaux-Ruff C, Neyton J, Paoletti P, Kieffer BL. Zinc alleviates pain through high-affinity binding to the NMDA receptor NR2A subunit. Nat Neurosci 2011; 14: 1017–22.

10. Safieh-Garabedian B, Poole S, Allchorne A, Kanaan S, Saade N, Woolf CJ. Zinc reduces the hyperalgesia and upregulation of NGF and IL-1 beta produced by peripheral inflammation in the rat. Neuropharmacology 1996; 35: 599–603.

11. Liu T, Walker JS, Tracey DJ. Zinc alleviates thermal hyperalgesia due to partial nerve injury. Neuroreport 1999; 10: 1619–23.

12. Jo SM, Danscher G, Schroder HD, Suh SW. Depletion of vesicular zinc in dorsal horn of spinal cord causes increased neuropathic pain in mice. Biometals 2008; 21: 151–8.

13. Luo J, Bavencoffe A, Yang P, Feng J, Yin S, Qian A, Yu W, Liu S, Gong X, Cai T, Walters ET, Dessauer CW, Hu H. Zinc Inhibits TRPV1 to Alleviate Chemotherapy-Induced Neuropathic Pain. J Neurosci 2018; 38: 474-83.
14. Haji Gholami A, Ansari H, Fardani F. Investigating the Effect of Zinc on the Prevention of Acute Peripheral Neuropathy in Cancer Patients Treated with Taxanes. Adv Biomed Res 2022; 11: 61.
15. Kanda Y. Investigation of the freely available easy-to-use software 'EZR' for medical statistics. Bone Marrow Transplant 2013; 48: 452-8.
16. Cheng HL, Lopez V, Lam SC, Leung AKT, Li YC, Wong KH, Au JSK, Sundar R, Chan A, De Ng TR, Suen LKP, Chan CW, Yorke J, Molassiotis A. Psychometric testing of the Functional Assessment of Cancer Therapy/Gynecologic Oncology Group-Neurotoxicity (FACT/GOG-Ntx) subscale in a longitudinal study of cancer patients treated with chemotherapy. Health Qual Life Outcomes 2020; 18: 246.
17. Calhoun EA, Welshman EE, Chang CH, Lurain JR, Fishman DA, Hunt TL, Cella D. Psychometric evaluation of the Functional Assessment of Cancer Therapy/Gynecologic Oncology Group-Neurotoxicity (Fact/GOG-Ntx) questionnaire for patients receiving systemic chemotherapy. Int J Gynecol

Cancer 2003; 13: 741-8.

18. Brundage MD, Pater JL, Zee B. Assessing the reliability of two toxicity scales: implications for interpreting toxicity data. J Natl Cancer Inst 1993; 85: 1138-48.

19. Tan AC, McCrary JM, Park SB, Trinh T, Goldstein D. Chemotherapy-induced peripheral neuropathy-patient-reported outcomes compared with NCI-CTCAE grade. Support Care Cancer 2019; 27: 4771–7.

20. Le-Rademacher J, Kanwar R, Seisler D, Pachman DR, Qin R, Abyzov A, Ruddy KJ, Banck MS, Lavoie Smith EM, Dorsey SG, Aaronson NK, Sloan J, Loprinzi CL, Beutler AS. Patient-reported (EORTC QLQ-CIPN20) versus physician-reported (CTCAE) quantification of oxaliplatin- and paclitaxel/carboplatin-induced peripheral neuropathy in NCCTG/Alliance clinical trials. Support Care Cancer 2017; 25: 3537–44.

21. Wang DS, Wang ZQ, Chen G, et al. Phase III randomized, placebo-controlled, double-blind study of monosialotetrahexosylganglioside for the prevention of oxaliplatin-induced peripheral neurotoxicity in stage II/III colorectal cancer. Cancer Med 2020; 9: 151-9.

22. Roberts JA, Jenison EL, Kim K, Clarke-Pearson D, Langleben A. A randomized,

multicenter, double-blind, placebo-controlled, dose-finding study of ORG 2766 in the prevention or delay of cisplatin-induced neuropathies in women with ovarian cancer. Gynecol Oncol 1997; 67: 172–7.

23. Koeppen S, Verstappen CC, Korte R, Scheulen ME, Strumberg D, Postma TJ, Heimans JJ, Hujigens PC, Kiburg B, Renzing-Kohler K, Diener HC. Lack of neuroprotection by an ACTH (4-9) analogue. A randomized trial in patients treated with vincristine for Hodgkin's or non-Hodgkin's lymphoma. J Cancer Res Clin Oncol 2004; 130: 153–60.

24. Gewandter JS, Mohile SG, Heckler CE, Ryan JL, Kirshner JJ, Flynn PJ, Hopkins JO, Morrow GR. A phase III randomized, placebo-controlled study of topical amitriptyline and ketamine for chemotherapy-induced peripheral neuropathy (CIPN): a University of Rochester CCOP study of 462 cancer survivors. Support Care Cancer 2014; 22: 1807–14.

25. Najafi S, Heidarali Z, Rajabi M, Omidi Z, Zayeri F, Salehi M, Haghighat S. Lithium and preventing chemotherapy-induced peripheral neuropathy in breast cancer patients: a placebo-controlled randomized clinical trial. Trials 2021; 22: 835.

26. Guo Y, Jones D, Palmer JL, Forman A, Dakhil SR, Velasco MR, Weiss M, Gilman

P, Mills GM, Noga SJ, Eng C, Overman MJ, Fisch MJ. Oral alpha-lipoic acid to prevent chemotherapy-induced peripheral neuropathy: a randomized, double-blind, placebo-controlled trial. Support Care Cancer 2014; 22: 1223–31.

27. Werida RH, Elshafiey RA, Ghoneim A, Elzawawy S, Mostafa TM. Role of alpha-lipoic acid in counteracting paclitaxel- and doxorubicin-induced toxicities: a randomized controlled trial in breast cancer patients. Support Care Cancer 2022; 30: 7281-92.

28. Gedlicka C, Scheithauer W, Schull B, Kornek GV. Effective treatment of oxaliplatin-induced cumulative polyneuropathy with alpha-lipoic acid. J Clin Oncol 2002; 20: 3359–61.

29. Leal AD, Qin R, Atherton PJ, Haluska P, Behrens RJ, Tiber CH, Watanaboonyakhet P, Weiss M, Adams PT, Dockter TJ, Loprinzi CL. North Central Cancer Treatment Group/Alliance trial N08CA-the use of glutathione for prevention of paclitaxel/carboplatin-induced peripheral neuropathy: a phase 3 randomized, double-blind, placebo-controlled study. Cancer 2014; 120: 1890–7.

30. Loprinzi CL, Qin R, Dakhil SR, Fehrenbacher L, Flynn KA, Atherton P, Seisler D, Qamar R, Lewis GC, Grothey A. Phase III randomized, placebo-controlled,

double-blind study of intravenous calcium and magnesium to prevent oxaliplatin-induced sensory neurotoxicity (N08CB/Alliance). J Clin Oncol 2014; 32: 997-1005.

31. Wesselink E, Winkels RM, van Baar H, Geijsen AJ, van Zutphen M, van Halteren HK, Hansson BM, Radema SA, de Wilt JH, Kampman E, Kok DE. Dietary Intake of Magnesium or Calcium and Chemotherapy-Induced Peripheral Neuropathy in Colorectal Cancer Patients. Nutrients 2018; 10: 398.
32. Kottscade LA, Sloan JA, Mazurezek MA, Johnson DB, Murphy BP, Rowland KM, Smith DA, Berg AR, Stella PJ, Loprinzi CL. The use of vitamin E for the prevention of chemotherapy-induced peripheral neuropathy: results of a randomized phase III clinical trial. Support Care Cancer 2011; 19: 1769–77.
33. Chen J, Shan H, Yang W, Zhang J, Dai H, Ye Z. Vitamin E for the Prevention of Chemotherapy-Induced Peripheral Neuropathy: A meta-Analysis. Front Pharmacol 2021; 12: 684550.
34. Schloss JM, Colosimo M, Airey C, Masci P, Linnane AW, Vitetta L. A randomised, placebo-controlled trial assessing the efficacy of an oral B group vitamin in preventing the development of chemotherapy-induced peripheral neuropathy (CIPN). Support Care Cancer 2017; 25: 195-204.

35. Smith EM, Pang H, Cirrincione C, Fleishman S, Paskett ED, Ahles T, Bressler LR, Fadul CE, Knox C, Le-Lindqwister N, Gilman PB, Shapiro CL. Effect of duloxetine on pain, function, and quality of life among patients with chemotherapy-induced painful peripheral neuropathy: a randomized clinical trial. JAMA 2013; 309: 1359–67.
36. Pujade-Lauraine E, Wagner U, Aavall-Lundqvist E, et al. Pegylated liposomal Doxorubicin and Carboplatin compared with Paclitaxel and Carboplatin for patients with platinum-sensitive ovarian cancer in late relapse. J Clin Oncol 2010; 28: 3323-9.
37. Takemoto S, Ushijima K, Honda K, Wada H, Terada A, Imaishi H, Kamura T. Precise evaluation of chemotherapy-induced peripheral neuropathy using the visual analogue scale: a quantitative and comparative analysis of neuropathy occurring with paclitaxel-carboplatin and docetaxel-carboplatin therapy. Int J Clin Oncol 2012; 17: 367-72.
38. Greenwald MK, Ruterbusch JJ, Beebe-Dimmer JL, Simon MS, Albrecht TL, Schwartz AG. Risk of incident claims for chemotherapy-induced peripheral neuropathy among women with breast cancer in a Medicare population. Cancer 2019; 125: 269-77.

39. Sahenk Z, Barohn R, New P, Mendell JR. Taxol neuropathy. Electrodiagnostic and sural nerve biopsy findings. Arch Neurol 1994; 51: 726–9.
40. Hara T, Chiba T, Abe K, Makabe A, Ikeno S, Kawakami K, Utsunomiya I, Hama T, Taguchi K. Effect of paclitaxel on transient receptor potential vanilloid 1 in rat dorsal root ganglion. Pain 2013; 154: 882–9.
41. Caterina MJ, Leffler A, Malmberg AB, Martin WJ, Trafton J, Petersen-Zeitz KR, Koltzenburg M, Basbaum AI, Julius D. Impaired nociception and pain sensation in mice lacking the capsaicin receptor. Science 2000; 288: 306–13.
42. Bautista DM, Jordt SE, Nikai T, Tsuruda PR, Read AJ, Poblete J, Yamoah EN, Basbaum AI, Julius D. TRPA1 mediates the inflammatory actions of environmental irritants and proalgesic agents. Cell 2006; 124: 1269–82.
43. Nagy I, Friston D, Valente JS, Torres Perez JV, Andreou AP. Pharmacology of the capsaicin receptor, transient receptor potential vanilloid type-1 ion channel. Prog Drug Res 2014; 68: 39-76.
44. Simone DA, Baumann TK, LaMotteRH. Dose-dependent pain and mechanical hyperalgesia in humans after intradermal injection of capsaicin. Pain 1989; 38: 99-107.
45. Witting N, Svensson P, Gottrup H, Arendt-Nielsen L, Jensen TS. Intramuscular

and intradermal injection of capsaicin: a comparison of local and referred pain. Pain 2000; 84: 407-12.

46. Xu Q, Zhang XM, Duan KZ, Gu XY, Han M, Liu BL, Zhao ZQ, Zhang YQ. Peripheral TGF-β1 signaling is a critical event in bone cancer-induced hyperalgesia in rodents. J Neurosci 2013; 33: 19099–111.

47. Facer P, Casula MA, Smith GD, Benham CD, Chessell IP, Bountra C, Sinisi M, Birch R, Anand P. Differential expression of the capsaicin receptor TRPV1 and related novel receptors TRPV3, TRPV4 and TRPM8 in normal human tissues and changes in traumatic and diabetic neuropathy. BMC Neurol 2007; 7: 11.

48. Boyette-Davis JA, Walters ET, Dougherty PM. Mechanisms involved in the development of chemotherapy-induced neuropathy. Pain Manag 2015; 5: 285–96.

49. Lee BE, Choi BY, Hong DK, Kim JH, Lee SH, Kho AR, Kim H, Choi HC, Suh SW. The cancer chemotherapeutic agent paclitaxel (Taxol) reduces hippocampal neurogenesis via down-regulation of vesicular zinc. Sci Rep 2017; 7: 11667.

50. Hoppe C, Kutschan S, Dörfler J, Büntzel J, Büntzel J, Huebner J. Zinc as a complementary treatment for cancer patients: a systematic review. Clin Exp Med 2021; 21; 297-313.

51. Abdelrahman KM, Hackshaw KV. Nutritional Supplements for the Treatment of Neuropathic Pain. Biomedicines 2021; 9: 674.

52. Bastow M, Kriedt CL, Baldassare J, Shah M, Klein C. Zinc is a potential therapeutic for chemoresistant ovarian cancer. J Exp Ther Oncol 2011; 9: 175-81.

53. Yin JL, Tao T, Wen ZY, Wang R, Sun MH, Gao C, Chang YJ, Yan S, Qin X, Zhao YH, Wang L, Gao S. Association between pre-diagnostic dietary copper, zinc, and copper-to-zinc ratio and severity of ovarian cancer. Front Nutr 2022; 9: 1003675.

54. Zhang P, Li Y, Tang X, Guo R, Li J, Chen YY, Guo H, Su J, Sun L, Liu Y. Zinc enhances chemosensitivity to paclitaxel in PC-3 prostate cancer cells. Oncol Rep 2018; 40: 2269-77.

55. Xue YN, Yu BB, Liu YN, Guo R, Li JL, Zhang LC, Su J, Sun LK, Li Y. Zinc promotes prostate cancer cell chemosensitivity to paclitaxel by inhibiting epithelial-mesenchymal transition and inducing apoptosis. Prostate 2019; 79: 647-56.

56. Gelbard A. Zinc in cancer therapy revisited. Isr Med Assoc J 2022; 24: 258-62.

57. Hesketh JE. Zinc-stimulated microtubule assembly and evidence for zinc

binding to tubulin. Int J Biochem 1982; 14: 983-90.

58. Tornabene D, Bini P, Gastaldi M, Vegezzi E, Asteggiano C, Marchioni E, Diamanti L. Neurological complications due to copper deficiency in the context of Wilson disease treatment: a case report with long-term follow-up and review of the literature. Neurol Sci 2024; 45: 987-96.

59. Nyrop KA, Deal AM, Reeder-Hayes KE, et al. Patient-reported and clinician-reported chemotherapy-induced peripheral neuropathy in patients with early breast cancer: Current clinical practice. Cancer 2019; 125: 2945-54.

Table 1：対象患者一覧

Patient No.	Age (years)	Primary cancer	FIGO stage	MaxG (CTCAE v5.0)	PreZn (μg/dL)	MinZn (μg/dL)	MinZn/PreZn Ratio	Number of TC cycles	BMI (kg/m^2)	History of diabetes	HbA1c (%)	eGFR (ml/min/1.73m^2)
1	72	Uterine	1	1	93	99	1.07	1 *	25.6	+	6.5	64.1
2	61	Uterine	4	1	75	75	1	6	22.0		5.7	83.1
3	53	Uterine	1	1	75	65	0.87	3	18.5		5.4	81.7
4	64	Uterine	1	1	94	93	0.99	3	26.6		5.8	49.4
5	31	Ovarian	1	1	81	74	0.91	6 *	18.1		5.4	85.7
6	66	Peritoneal	3	1	50	47	0.94	6	22.8	+	7.1	144.9
7	59	Peritoneal	3	1	67	60	0.90	5	22.5		5.9	72.5
8	58	Uterine	3	2	73	54	0.74	6	20.2		5.5	79.6
9	59	Uterine	1	2	69	69	1	6	24.9		6.0	73.4
10	75	Peritoneal	3	2	56	43	0.77	6	24.2	+	8.2	76.8
11	58	Uterine	2	3	81	74	0.91	3 **	26.1	+	6.4	93.4
12	62	Uterine	2	3	79	57	0.72	6 *	20.0		5.5	76.7
13	78	Ovarian	4	4	42	34	0.81	3 **	25.5		5.9	73.1

* : dose reduction or/and discontinuation not caused by CIPN, ** : dose reduction and discontinuation caused by CIPN

Table 2: CIPN 治療に関する臨床試験

References	Treatment for CIPN	Trials type	Sample size	Evaluation method	Endpoints	Chemotherapy	Outcomes
Wang 2020[23]	GM1, iv	RCT	196	NCI-CTCAE	Rate of grade 2 or worse cumulative neurotoxicity	FOLFOX	n.s.<br>(GM1: 33.7% vs placebo: 31.6%, P = 0.76)
Roberts 1997[24]	Hexapeptide of ACTH, iv	RCT	196	VPT	Percentage change in VPT	Cisplatin	n.s.
Koeppen 2004[25]	ACTH analogue, iv	RCT	147	13-item questionnaire	Neuropathy-free interval (the first occurrence of bilateral paresthesias)	Vincristine	n.s.
Gewandter 2014[26]	2% ketamine +<br>4% amitriptyline cream	RCT	462	NRS	6-week NRS	Taxane 53%	n.s. (P=0.363)
Najafi 2021[27]	Lithium, po	RCT	36	decision by oncologist	Frequency of symptoms	Taxane	n.s. (P=0.352)
Guo 2014[28]	ALA, po	RCT	243	FACT/GOG-Ntx	FACT/GOG-Ntx scores at 24 weeks	Cisplatin or Oxaliplatin	n.s.
Werida 2022[29]	ALA, po	RCT	64	NCI-CTCAE	Grade comparison every three weeks	Paclitaxel	Significant improvement after the end of 9th and 12th weeks of paclitaxel intake (P=0.039)
Leal 2014[31]	Glutathione, iv	RCT	185	EORTC QLQ-CIPN20, NCI-CTCAE	Subscale of the EORTC QLQ-CIPN20 during the first 6 cycles of chemotherapy	TC	n.s.<br>(EORTC QLQ-CIPN20: P=0.21, NCI-CTCAE: P=0.449)
Loprinzi 2014[32]	Calcium + Magnesium, iv	RCT	353	EORTC QLQ-CIPN20	Cumulative neurotoxicity	FOLFOX	n.s.
Wesselink 2018[33]	Calcium + Magnesium, po	Prospective cohort study	196	EORTC QLQ-CIPN20	The score before surgery, during chemotherapy and six months after chemotherapy	Oxaliplatin 85%	Dietary intake of magnesium during chemotherapy was associated with the prevalence of CIPN (PR 0.53, 95% CI 0.32, 0.90), and a higher dietary intake of magnesium was associated with less severe symptoms of CIPN
Kottscade 2011[34]	Vitamin E, po	RCT	207	NCI-CTCAE	Incidence of grade 2+	Taxane 58%<br>Oxaliplatin 26%	n.s.<br>(vitamin E: 34% vs placebo: 29%, P=0.43))
Chen 2021[35]	Vitamin E, po	meta-analysis	486	NSS, NDS, FGS	Incidense of CIPN	Taxane, Cisplatine, Oxaliplatin, others	Significantly reduced the incidence of CIPN<br>(overall RR = 0.55, 95% CI: 0.36, 0.85, P=0.007)
Schloss 2017[36]	Vitamin B, po	RCT	71	TNS	TNS assessed by an independent neurologist	Taxane, Oxaliplatin, Vincristine	n.s. in CIPN incidence (P=0.73) but reduced severity<br>(12 weeks, P=0.03; 24 weeks, P=0.005; 36 weeks, P=0.021)

GM1: monosialotetrahexosylganglioside, RCT: randomized controlled trial, FOLFOX: fluorouracil+leucovorin+oxaliplatin, n.s.: no significant differences, ACTH: adrenocorticotropic hormone, VPT: vibration perception threshold, NRS: numeric rating scale, ALA: alpha-lipoic acid, NSS: neurological symptom score, NDS: neurological disability score, FGS: Hughes' functional grading scale, TNS: total neuropathy score

Figure 1　血清亜鉛値および TC 療法施行サイクル数と MaxG の相関関係

MinZn/PreZn 比と MaxG に有意な負の相関を認め，CIPN が重症であれば血清亜鉛値の低下が多い結果となった（r=-0.557，p=0.048）．以下は MaxG との間に相関性を認めなかった．b) PreZn (r=-0.403，p=0.172)，c) MinZn (r=-0.525，p=0.066)，d) TC 療法施行サイクル数（r=-0.054，p=0.861)

Figure 1a

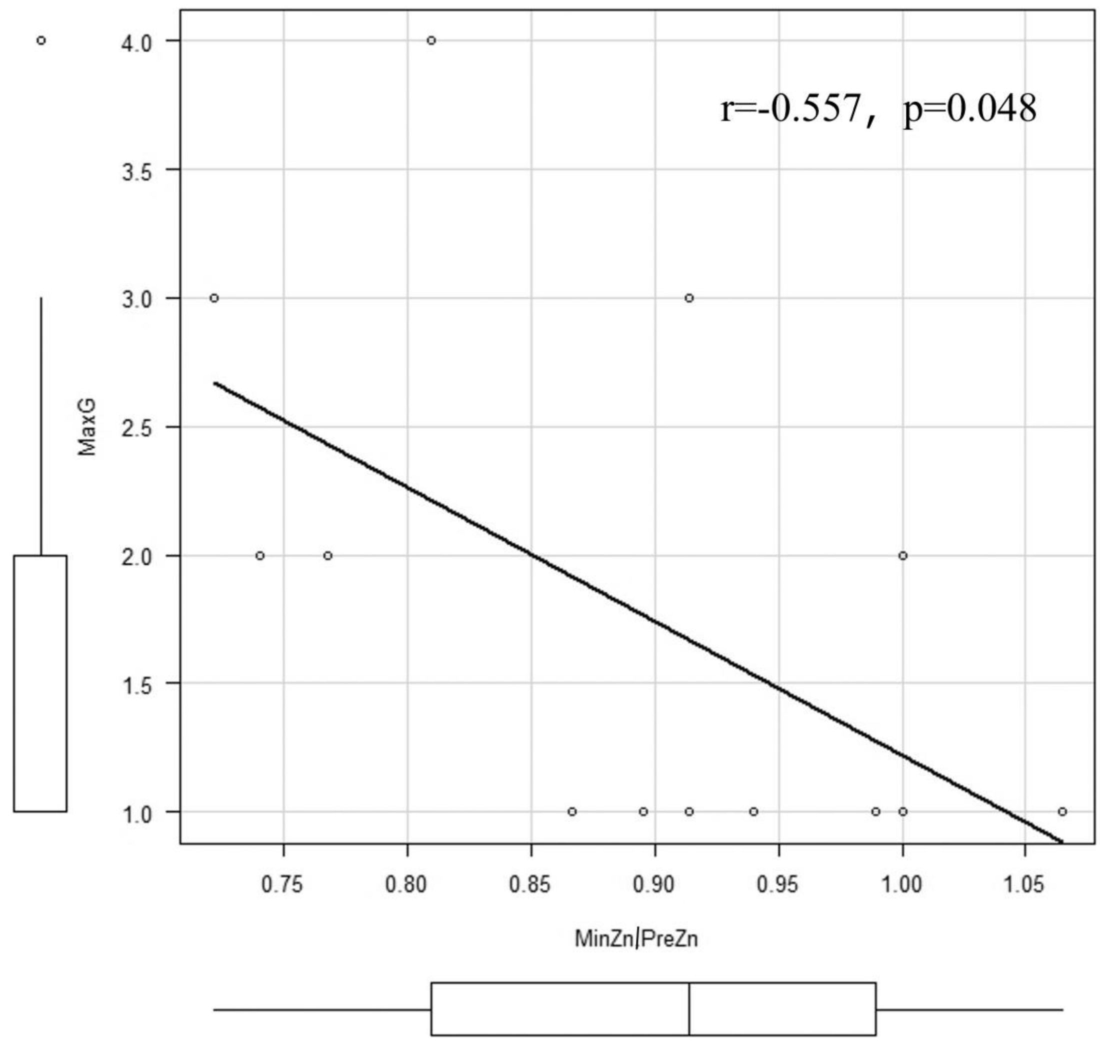

r=-0.557, p=0.048
MaxG
MinZn/PreZn
4.0
3.5
3.0
2.5
2.0
1.5
1.0
0.75
0.80
0.85
0.90
0.95
1.00
1.05

Figure 1b

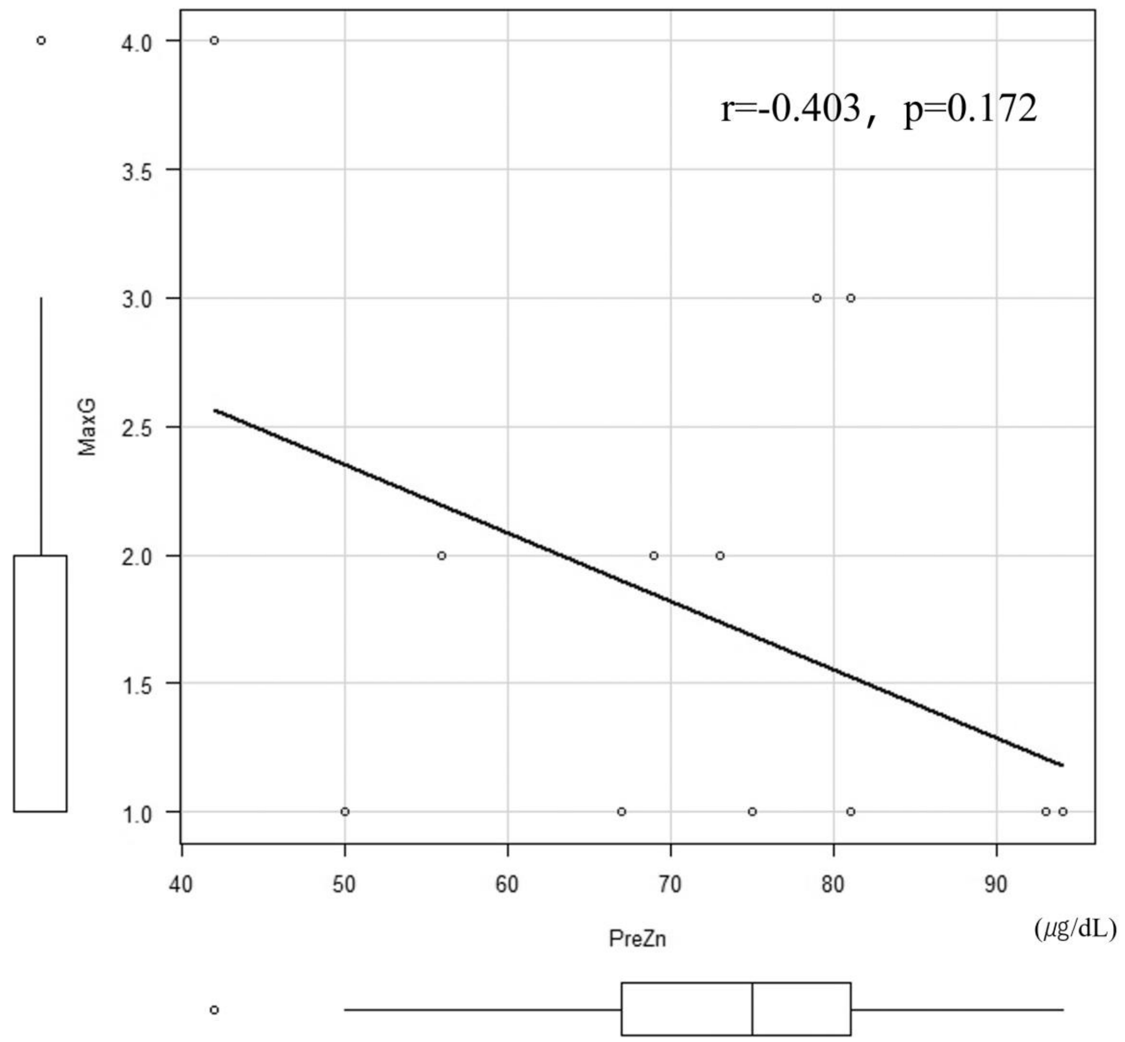

Figure 1c

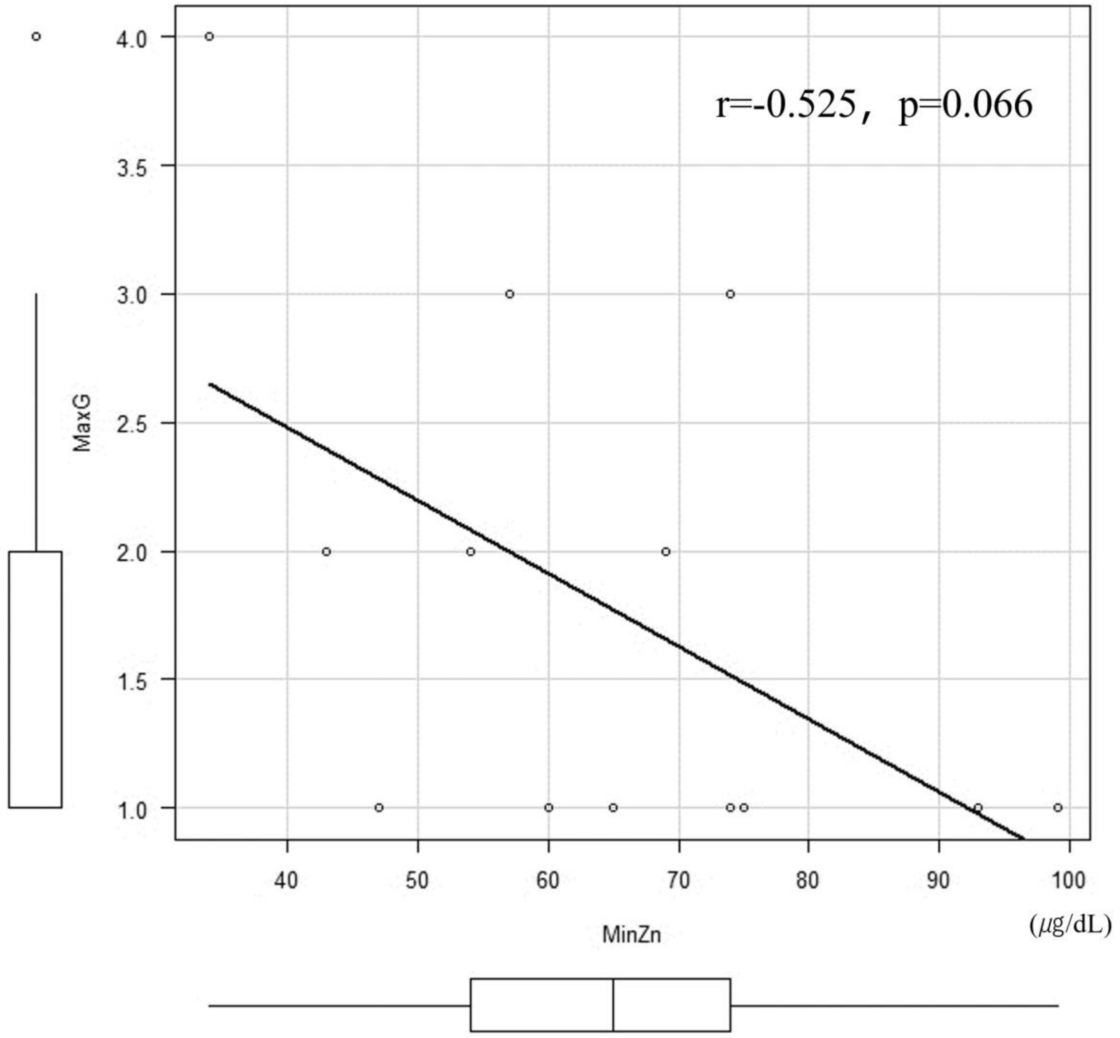
r=-0.525, p=0.066
4.0
3.5
3.0
2.5
2.0
1.5
1.0
MaxG
40
50
60
70
80
90
100
(μg/dL)
MinZn

Figure 1d

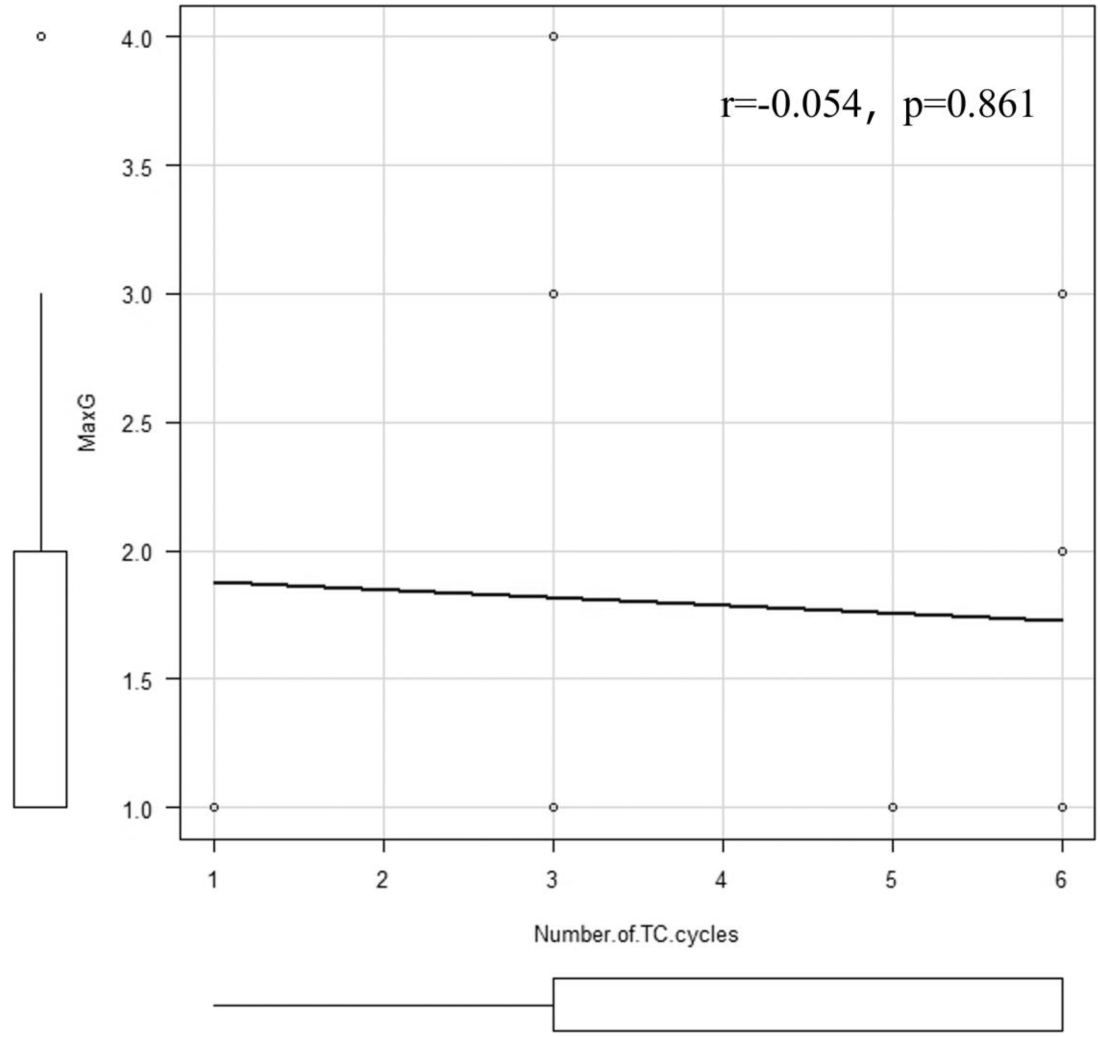
r=-0.054, p=0.861
MaxG
4.0
3.5
3.0
2.5
2.0
1.5
1.0
1
2
3
4
5
6
Number.of.TC.cycles

Figure 2　MaxG 1（MaxG 1 群）と MaxG 2 以上（MaxG 2+群）に分類した場合の臨床学的因子による検討

以下の臨床学的因子は CIPN の重症度に影響を与えなかった．a) 年齢 (p=0.667)，b) BMI (p=0.628)，c) HbA1c (p=0.566)，d) eGFR (p=1.000)

Figure 2a

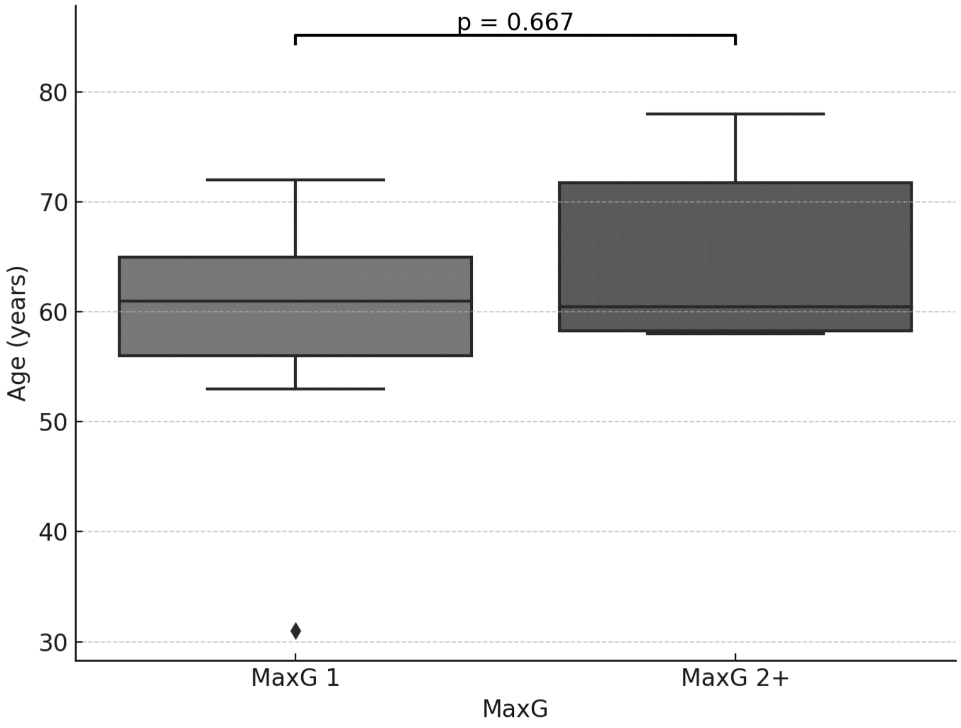

Figure 2b

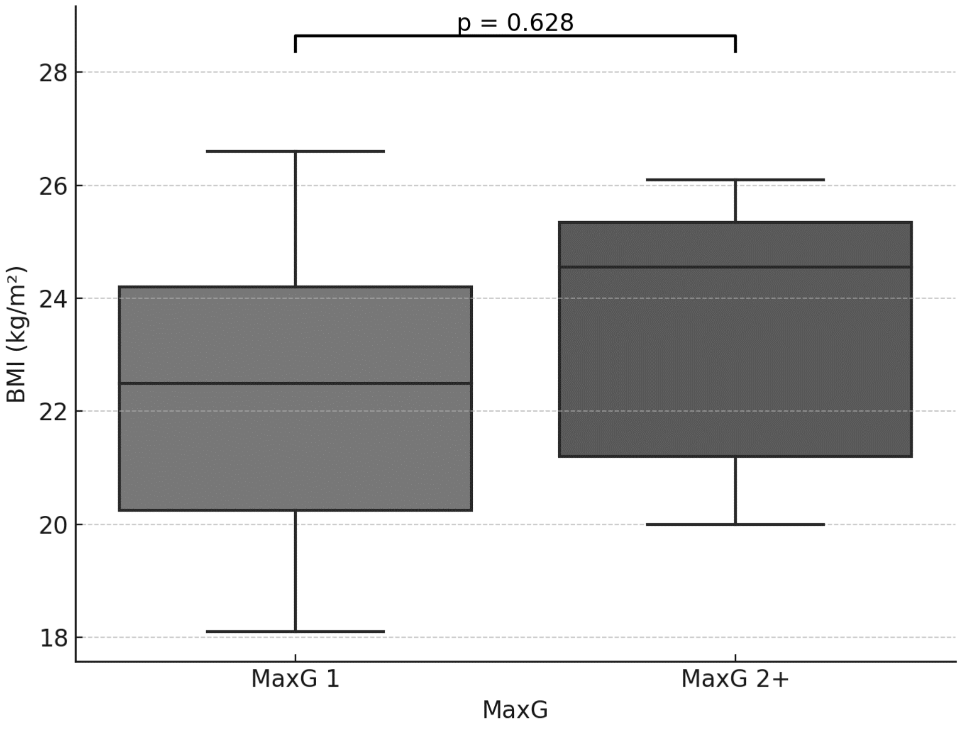

Figure 2c

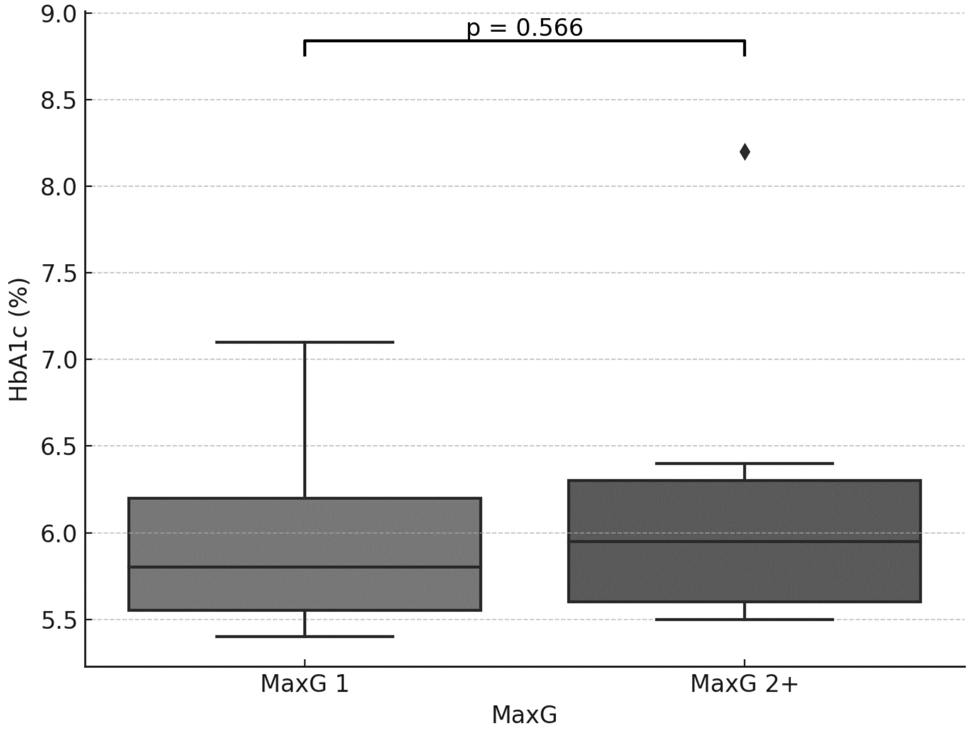

Figure 2d

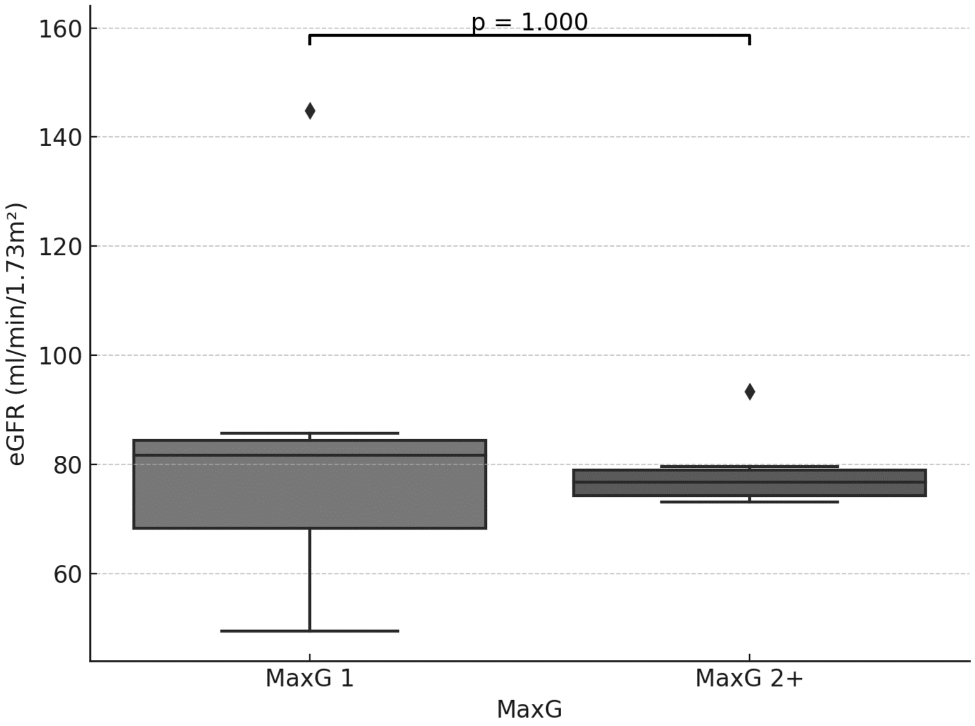

Figure 3　TC 療法施行サイクル毎の血清亜鉛値と CIPN グレード平均の推移

　TC 療法施行前と TC 療法施行サイクル毎の血清亜鉛値を，MaxG 1（薄いグレー）と MaxG 2+（濃いグレー）に分類し箱ひげ図を並記した．MaxG 2+群で血清亜鉛値が低値である傾向がみられ，サイクル 4 では MaxG 2+群が MaxG 1 群と比較して有意に血清亜鉛値が低値であった（＊：p=0.026）.

　右上段に MaxG 2+群における TC 療法施行サイクル毎の CIPN グレード平均値を折れ線グラフで記載した．TC 療法施行サイクル数増加に伴って CIPN グレードは増加したが，3 サイクル目以降はプラトーとなった.

Figure 3

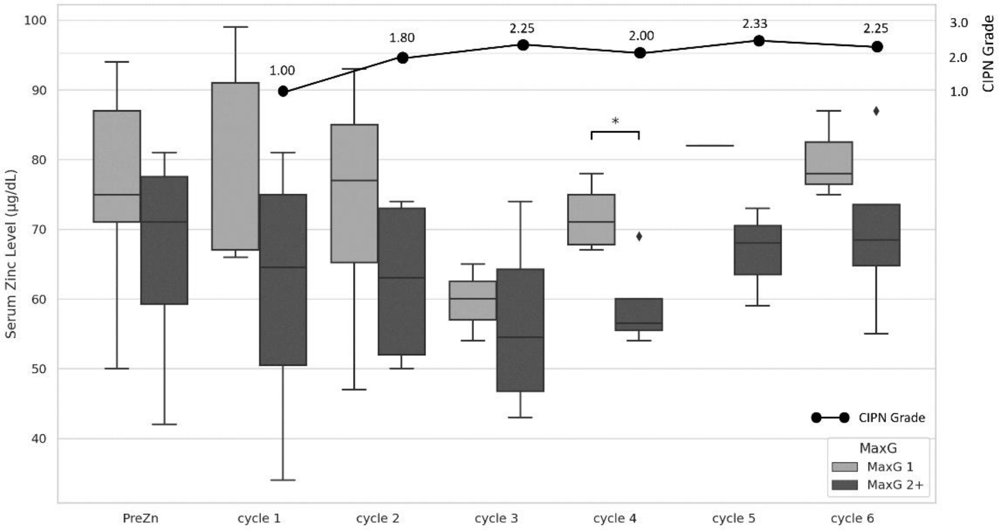

Serum Zinc Level (µg/dL)
CIPN Grade
1.00
1.80
2.25
2.00
2.33
2.25
*
CIPN Grade
MaxG
MaxG 1
MaxG 2+
PreZn
cycle 1
cycle 2
cycle 3
cycle 4
cycle 5
cycle 6
